# Identification and validation of tumor microenvironment-related therapeutic targets in gastric cancer using integrated multi-omics and molecular docking approaches

**DOI:** 10.3389/fbinf.2025.1654326

**Published:** 2025-12-10

**Authors:** Mohamed Kalith Oli M., Jafar Ali Ibrahim Syed Masood

**Affiliations:** 1 School of Bio Sciences and Technology, Vellore Institute of Technology, Vellore, India; 2 Department of Quantum AI, School of Computer Science and Engineering, Vellore Institute of Technology, Vellore, India

**Keywords:** gastric cancer, differentially expressed genes, extracellular matrix, molecular docking, therapeutic targets, personalized medicine

## Abstract

**Introduction:**

With increased drug resistance and tumor heterogeneity accounting for limited therapeutic strategies, gastric cancer remains one of the major causes of cancer-related mortality around the globe. Targeting the components of the tumor microenvironment (TME) has become a promising therapeutic strategy due to their crucial roles in cancer cell proliferation, progression, and metastasis. One of the limitations of the previously identified therapeutic targets is their limited applicability to a broader patient population.

**Methods:**

This study aims to identify (TME)-related therapeutic targets using an integrated bioinformatics and molecular docking approach that involves a larger number of datasets to cover a broader cohort of gastric cancer patients. It analyzed multiple publicly available transcriptomic datasets using Robust Rank Aggregation (RRA) meta-analysis and Weighted Gene Co-expression Network Analysis (WGCNA) to identify significant hub genes. Furthermore, protein-protein interaction (PPI) network analyses, conducted using multiple methods such as Cytohubba topology analysis and ClusterONE module analysis, refined the potential therapeutic candidates. Functional enrichment analyses were performed to identify vital genes involved in TME interactions and ECM remodeling.

**Results:**

The enriched genes were validated for their significant dysregulation in the Cancer Genome Atlas gastric adenocarcinoma dataset (TCGA-STAD) and three independent GEO datasets to ensure differential expression across distinct cohorts. Genes with consistent dysregulation were used in survival analyses across TCGA and two GEO datasets to prioritize hub genes with prognostic significance. Finally, a targeted literature survey ensured the exclusion of previously targeted genes, and molecular docking analyses conducted using phytocompounds identified potential therapeutic leads with strong affinities for the identified targets.

**Discussion:**

This integrated approach revealed notable, promising targets in the TME and natural compounds for developing potential personalized therapeutic strategies in gastric cancer.

## 1 Introduction

Gastric cancer (GC) is one of the most prevalent malignancies, which ranks fifth in both incidence and mortality, with more than 968,000 new cases and 660,000 deaths around the globe (Bray et al., 2024). Endoscopy with biopsy remains the standard diagnostic procedure for gastric cancer, as it enables both visualization and histological confirmation (Vincze, 2023). Recent advancements in medical imaging, combined with the limited but evolving use of biomarkers, have improved the detection rates of gastric cancer. The treatment strategies for gastric cancer include surgical resection for the early stages and combinations of surgery, radiation therapy, and chemotherapy for the advanced stages, according to the molecular characteristics of the tumor (Mamun et al., 2024). With an increased understanding of GC pathogenesis mechanisms, targeted therapeutic options such as inhibiting HER2, EGFR, VEGF/VEGFR, and CLDN18.2 have become promising topics for research and clinical trials. Targeted therapeutic strategies inhibit the defective signaling pathways in cancer, thereby causing less damage to normal cells. Some of the widely tested targeted therapeutic strategies include targeting HER2 with trastuzumab or new agents like T-DXd (trastuzumab deruxtecan) and inhibiting angiogenesis by targeting VEGF/VEGFR with bevacizumab or ramucirumab (Javle et al., 2014; Ishii and Shitara, 2021). Another recently approved targeted therapy strategy is neutralizing the CLDN18.2 with the antibody zolbetuximab (Kubota and Shitara, 2024). Targeting EGFR with drugs like cetuximab and panitumumab combined with chemotherapy did not significantly improve patient survival in phase III clinical trials (Lordick et al., 2012; Waddell et al., 2012). The main challenges are that only a subset of gastric cancer patients express these targets, intratumor heterogeneity, and those who express these usually develop drug resistance (Luo et al., 2025). Recently, studies have identified numerous potential therapeutic targets by analyzing expression profiling datasets; however, only a small subset of the total datasets is generally selected. Thus, the chosen hub genes represent a small population of patients with GC (Wang R. et al., 2022; Hu et al., 2023; Wu et al., 2025). Therefore, the discovery of multiple novel therapeutic targets that many patients express could broaden the proportion of treatable gastric cancer cases and enable novel combination strategies for targeted therapy. Natural compounds have been considered important for cancer therapy due to their ability to regulate multiple cancer-related pathways and comparatively safe ADMET profiles (Nan et al., 2023). This study used various approaches to ensure that biologically significant targets were identified from the available GEO datasets. It utilized both microarray and RNA sequencing datasets that cover a considerable number of the gastric cancer population, integrated the aggregated differentially expressed genes from Robust Rank Aggregation (RRA) analysis with hits from tumor-correlated modules in Weighted Gene Co-expression Network Analysis (WGCNA), and used those genes for protein-protein interaction and enrichment analyses. This methodology ensured that the selected targets were expressed across a wide patient population and were pivotal to regulatory networks. Furthermore, to ensure the broad expression, the chosen targets were validated in TCGA and GEO datasets, followed by survival analyses to ensure that prognostically significant hub genes were chosen. Finally, the chosen targets were evaluated for interactions with natural compounds using molecular docking to identify potential therapeutic leads, thereby providing insights into their translational potential.

## 2 Materials and methods

### 2.1 Data sources and inclusion criteria

The Gene Expression Omnibus (GEO) database was used to retrieve expression profiling datasets using the search term ‘gastric cancer' (https://www.ncbi.nlm.nih.gov/geo/) (Barrett et al., 2012). The criteria for dataset selection are as follows:

i. Datasets from high-throughput sequencing and single-channel microarray experiments with proper gene annotations were included.
ii. Paired tumor and adjacent normal tissue datasets were selected with a minimum sample size of 6.
iii. Experiments involving non-coding RNA, as well as methylation profiling or studies involving metastatic samples and other conditions such as gastritis or cachexia, were excluded because they could introduce confounding variables that might affect the reliability of the results.

For validation of differential expression results and survival analyses, a large cancer genomics cohort from TCGA, as well as at least 2 external GEO datasets from geographically distinct populations, was obtained.

**FIGURE 1:**
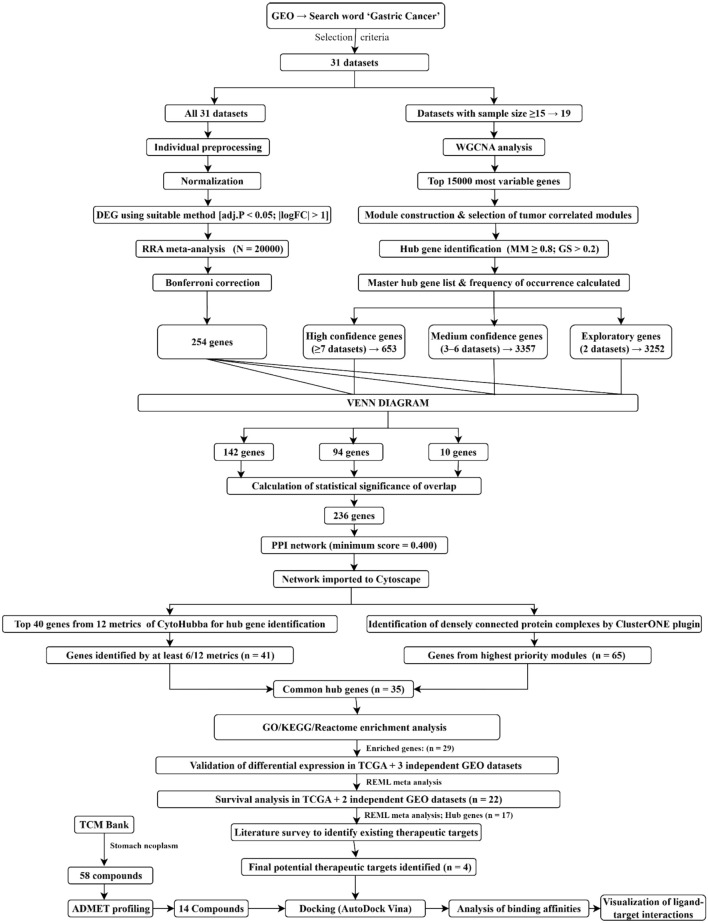
Integrative workflow used in this study to discover potential therapeutic targets and lead natural compounds. An integrative RRA meta-analysis and a WGCNA-based approach was utilized to identify hub genes. Protein-protein interaction network analysis, including Cytohubba metrics and ClusterONE, along with functional enrichment analyses, led to the selection of the final potential therapeutic targets. Furthermore, differential expression was independently validated in separate cohorts, followed by molecular docking analyses using active compounds from TCMBank to identify potential lead compounds and their interactions with the targets.

The integrative approach considered RNA sequencing experiments due to their high sensitivity and dynamic range. Additionally, it included extensive data obtained from microarray experiments to produce reliable results. Figure 1 depicts the workflow used in this study.

### 2.2 Differential expression analysis and meta-analysis using robust rank aggregation (RRA) algorithm

Selected datasets were preprocessed and normalized individually, and differentially expressed genes (DEGs) were assessed using methods such as limma, DeSeq2, and limma-voom (Law et al., 2014; Love et al., 2014; Ritchie et al., 2015). All analyses were conducted in R using Bioconductor packages. For each dataset, genes were given raw p-values, log2 fold changes, and adjusted p-values. All genes were sorted primarily by adjusted p-values and then by absolute log fold change to indicate statistical significance.

The ‘RobustRankAggreg’ package in R was used for the meta-analysis of DEG results, as it is useful for combining gene expression results from multiple datasets. Here, the RRA algorithm integrated and ranked the genes consistently across all datasets, ensuring that variation across different platforms was captured (Kolde et al., 2012). The parameter was set to N = 20,000 to include all the significant genes and reduce the noise. Furthermore, Bonferroni correction, as shown in Equation 2.1, was applied, and the genes with scores below this threshold were selected for further analysis.

$$\alpha_{corrected}=\frac{\alpha }{m}$$ (2.1)

$\alpha_{\text{corrected}}$ is the adjusted significance threshold

α is the original threshold

m is the number of tests (genes).

### 2.3 WGCNA analysis and hub gene identification

The WGCNA algorithm was applied to all datasets that met the minimum sample size of 15 to identify significant gene co-expression networks and their correlation with the tumor phenotype (Langfelder and Horvath, 2008). Each dataset was preprocessed and normalized individually using appropriate methods, and the top 15,000 most variable genes were selected for the analysis using the WGCNA package in R. For each dataset, sample clustering was performed to remove outliers, ensuring high sample quality. Appropriate soft-threshold powers were chosen from the scale-free topology fit plot where R2 crosses 0.8. This was followed by signed weighted gene co-expression network construction and the detection of modules through hierarchical clustering with the dynamic tree-cut method. Furthermore, highly similar modules were merged to refine the assignment of modules. After analyzing module-trait relationships, modules with significant correlations with tumor samples were selected for further analysis to capture robust biological signals from all the datasets. From the tumor-correlated modules with module trait p-value less than 0.05 of each dataset, genes with module membership (MM) greater than 0.8 and gene significance (GS) more than 0.2 were chosen as hub genes. Unique gene symbols of tumor-associated hub genes from each dataset were combined to create a master hub gene list and a presence-absence logical matrix to indicate the gene occurrences across the selected datasets. After calculating the frequency of occurrence, genes were divided into three confidence categories: high-confidence genes, which appear in 7 or more datasets; medium-confidence genes, which appear in 3–6 datasets; and exploratory genes, which appear in at least 2 datasets. Genes present only in one dataset were considered low-support genes and were excluded from further analysis.

### 2.4 Integration of meta-DEG and WGCNA results

A common background universe (U) was defined as the set of unique genes common to the ranked genes from the RRA analysis and to the genes considered in the WGCNA analysis. All gene lists, including the universe list, were mapped to HGNC symbols using limma, HGNChelper and org.Hs.eg.db and duplicates were removed (Oh et al., 2020; Carlson et al., 2019). Later, for each category (High, medium, and exploratory), 2 × 2 contingency tables were constructed to count.

i. Genes present in both the RRA list and the WGCNA category (a)
ii. Genes present in the WGCNA category but not in the RRA list (b)
iii. Genes present in the RRA list but not in the WGCNA category (c)
iv. Remaining genes from the background (d); (|U| - (a+b + c))

Then, a one-sided Fisher’s exact test was applied as in Equation 2.2 to determine if the observed overlap is more than what would be expected by chance (Fisher, 1922). Along with the p-value, an odds ratio representing the strength of association between WGCNA category membership and presence in the RRA list was reported. Furthermore, a representation factor, calculated as the observed overlap divided by the expected overlap under random sampling, was calculated; values greater than 1 indicated over-representation. P-values across the three categories were adjusted by Benjamini-Hochberg false discovery rate (BH-FDR), and only those categories with significant over-representation (FDR<0.05) were chosen for downstream analyses (Benjamini and Hochberg, 1995).

$$p_{\text{greater}}=\sum_{i=a}^{\min\left(\left|T\right|,\left|RRA\right|\right)}\frac{\left(\begin{array}{c} \left|RRA\right|\\ i \end{array}\right)\left(\begin{array}{c} \left|U\right|-\left|RRA\right|\\ \left|T\right|-i \end{array}\right)}{\left(\begin{array}{c} \left|U\right|\\ \left|T\right| \end{array}\right)}$$ (2.2)

U = Background universe

RRA = set of RRA hits

T = WGCNA tier

a = Observed overlap between the WGCNA tier and RRA hits

### 2.5 Protein-protein interaction network and prioritization of hub genes

Overlapping genes with statistical significance were analyzed for protein-protein interactions using the STRING (https://string-db.org/) and STITCH (http://stitch.embl.de/) databases (Kuhn et al., 2007; Szklarczyk et al., 2019). Interaction networks with a minimum score of 0.400 or medium confidence were imported to Cytoscape software (version 3.10.3) (Shannon et al., 2003). All 12 metrics, such as Maximal Clique Centrality (MCC), Degree, Betweenness, and Maximum Neighborhood Component (MNC) and others from the CytoHubba plugin were used to select the top 40 genes for prioritizing the hub genes. The genes identified consistently by at least 50% of the metrics were chosen as hub genes from Cytohubba (Chin et al., 2014). Simultaneously, the PPI network was evaluated for overlapping protein complexes using ClusterONEweb (https://paccanarolab.org/clusteroneweb/) with a minimum module size of 3, a minimum density of 0.3, a maximum overlap of 0.6, and a penalty of 2 for larger clusters (Baez et al., 2025). The results with p-values were downloaded, and the top modules were selected based on size, density, and p < 0.05. Furthermore, the common genes from both analyses were visualized using the VennDiagram (1.7.3) package in R for finalizing targets for enrichment analyses, such as Kyoto Encyclopedia of Genes and Genomes (KEGG) and Gene Ontology (GO) (Oliveros, 2007).

### 2.6 Functional enrichment analysis and hub gene selection

Enrichment analyses, including GO molecular function (MF), cellular component (CC), and biological process (BP), KEGG, and Reactome pathway enrichment analyses, were conducted using the clusterProfiler, ReactomePA packages, org.Hs.eg.db, AnnotationDbi, and enrichplot in R (Yu et al., 2012; Yu and He, 2016; Pagès et al., 2017; Guangchuang et al., 2023). The overlapping genes from the cluster and topology PPI analyses were considered as target genes, and the initial set of genes from the PPI network was chosen as the background. Gene symbols were mapped to their Entrez IDs using the bitr function of the Annotation DBI package. Enrichments were calculated using the mapped background, BH correction, and thresholds such as p. Adjust ≤0.05, q-value ≤0.20, minimum gene set size of 3, and a maximum of 200. Gene ratios, background ratios, and fold enrichment values were reported; dot plots were generated for top terms, and the terms with p.adjust ≤0.05, q-value ≤0.2, fold enrichment ≥2, and a minimum gene count of 3 were considered significant. The candidates that contributed to significant terms in functional enrichment analysis were prioritized for validation and survival analyses.

### 2.7 Validation of differential expression

To validate the differential expression of the chosen candidates, datasets from distinct clinical populations were chosen. It included TCGA-STAD (RNA seq) (Colaprico et al., 2016), GSE54129 (GPL570; China), GSE13911 (GPL570; Italy) (D’Errico et al., 2009) and GSE66229 (GPL570; Asian Cancer Research Group (ACRG cohort)) (Oh et al., 2018). These cohorts were not utilized in the discovery data and were preprocessed with platform-specific methods and mapped to HGNC gene symbols using org.Hs.eg.db and hgu133plus2.DB before validating the dysregulation of the selected genes (Carlson et al., 2016). For the TCGA cohort, STAR counts were imported, and samples with ambiguous labels from the clinical metadata and genes with zero total counts were removed, and duplicates were collapsed after mapping to HGNC symbols. Later, the RNA sequencing data were analyzed for differential expression with DESeq2, which called on unshrunken log2 fold-changes with BH-FDR. The raw CEL files of the GEO microarray cohorts were RMA normalized with affy, and for genes with multiple probes, the probe with the largest interquartile range (IQR) across samples was retained (Irizarry et al., 2003). They were tested with limma and log2FC, standard errors (SE), p, and FDR values were recorded. Regardless of the significance of each study, meta-analysis combined the log2FC ± SE for each gene from each cohort using a random-effects model (REML) using metafor (Viechtbauer, 2010). This meta-analysis reported heterogeneity (Q, inconsistency index I^2^, and τ^2^), two-sided p-values, pooled log2FC, and 95% confidence intervals. Furthermore, BH-FDR was applied to the selected genes, and the REML model was refitted by excluding one cohort at a time (Leave-one-out; LOO) to assess sensitivity. Genes with no changes in the pooled effect’s sign were labeled as stable under LOO. Genes were validated as differentially expressed if they had FDR ≤0.05 and a CI excluding 0 in the REML model, and had a consistent sign in the LOO analysis and p ≤ 0.05.

### 2.8 Survival analysis and candidate gene selection

Three independent gastric cancer cohorts were used to evaluate the effect of the selected genes on overall survival (OS): TCGA-STAD (RNA sequencing), GSE62254 (microarray), and GSE15459 (microarray) (Ooi et al., 2009; Cristescu et al., 2015). OS was measured as the time from diagnosis to death, with event 1 indicating death and 0 indicating censoring. For TCGA, STAR counts were collapsed to HGNC symbols, normalized, and transformed using the standard size factor normalization of DESeq2 (Variance Stabilizing Transformation, VST), and RMA normalization (affy) was used for microarray datasets. Gene expression was standardized to a mean of 0 and a standard deviation of 1 before modeling in each cohort. Kaplan-Meier (KM) plots were created for visualization using the top 25% vs. the bottom 25% of expression (Q4 vs. Q1) within the cohorts to avoid dilution around the median, and log-rank p-values, along with n-at-risk tables, were added to plots for all genes.

Cox proportional-hazards models were applied to all candidate genes in each cohort. Univariate models related the OS to the standard expression of each gene, followed by multivariate models that adjusted for clinical covariates. For TCGA, the multivariate models were adjusted for age and sex, whereas for the GEO cohorts, age, sex, and stage were adjusted. Across all the models, hazard ratio (HR), 95% CI, the Wald p-value, a global Schoenfeld test p-value that checks the proportional hazards, and model discrimination using Harrell’s c-index were recorded. Moreover, BH-FDR correction was applied to each cohort in both univariate and multivariate analyses. For cohorts with stage data, gene × stage interactions were evaluated with stage Ⅰ as the reference and stages Ⅱ, Ⅲ, and Ⅳ as contrasts, followed by BH-FDR correction across genes. Interactions were considered significant only if they had FDR ≤0.05. The univariate Cox HRs for each gene across all three cohorts were combined in a meta-analysis using metafor, with a random-effects REML model. This analysis reported pooled HRs with 95% CI, p-values, and BH-FDR testing across the genes, along with heterogeneity values (Q, I2, and τ2). Genes were considered significant in the meta-analysis if they had an HR > 1 in at least 2 of the 3 cohorts, indicating that their higher expression was associated with worse overall survival. The REML model was refitted by dropping one cohort at a time (LOO), and genes were considered stable under LOO if there was no loss of significance and sign changes. The final candidate genes with survival significance were selected if the pooled HR > 1 and the pooled FDR ≤0.05, if the HR > 1 in ≥2/3 cohorts with no sign changes in LOO.

### 2.9 Molecular docking analysis

For the final candidate genes, a targeted literature survey was conducted to exclude the already validated therapeutic targets. Only those genes without prior *in vitro* or *in silico* targeting were chosen as final therapeutic candidates. TCMBank was selected as the compound library for docking, and the phytocompounds for the search term ‘stomach neoplasm’ were retrieved for ADMET profiling (Lv et al., 2023). The drug-likeness, pharmacochemical, and pharmacokinetic properties were evaluated using SWISS ADME (Daina et al., 2017). Toxicity profiling was performed using ProTox-III, and compounds with acceptable ADMET profiles were shortlisted; their 3D structures were downloaded from PubChem (Banerjee et al., 2024; Kim et al., 2025). The shortlisted phytocompounds were optimized and converted to the required PDBQT format using OpenBabel (O’Boyle et al., 2011).

i. When experimentally validated structures were available, 3D coordinates with high resolution were downloaded from the RCSB PDB (Berman, 2000). The coordinates were cleaned and protonated using MGL Tools.
ii. In the absence of experimentally validated structures, AlphaFold models were retrieved corresponding to the human Uniprot entries of the targets (UniProt Consortium, 2018). Regions of low confidence (pLDDT<50) were removed using PyMOL, and missing loops were remodeled using SWISS-MODEL (Schrodinger, 2015; Waterhouse et al., 2018; Jumper et al., 2021). The edited models were saved and used for cavity prediction.

Target preparations were performed using MGL tools, and primary binding pockets of the finalized receptors were predicted using the PrankWeb, which provided the grid center coordinates for further docking analysis (Polák et al., 2025). To maintain consistency across all receptor sets, no bound metal ions or cofactors were retained in any of the receptor files. AutoDock Vina was used to perform the docking analysis, where the grid of each target was centered on the Prankweb-predicted primary binding pocket (Trott and Olson, 2010). A uniform cubical box of 24 × 24 × 24 Å, exhaustiveness of 32, energy range of 5 kcal mol^-1^ and 20 modes were used consistently across all docking runs, and the highest-scoring Vina pose of all the ligands was retained. The targets and the ligands were docked in triplicate with different random seeds to confirm that the binding affinities did not vary much, and the top-scoring ligand pose was visualized with the corresponding target proteins in Biovia Discovery Studio Visualizer (v21.1.0.20298) (BIOVIA, 2021).

## 3 Results

### 3.1 Dataset selection

A total of 31 publicly available datasets that matched the inclusion criteria (discussed in Section 2.1) were retrieved from the GEO website for the comparative transcriptomic analysis. Of these, eighteen datasets were submitted from single-channel microarray experiments, and thirteen were generated from high-throughput RNA sequencing experiments. Across the chosen datasets, 927 tumor samples and 919 matched adjacent normal stomach tissues were included, providing a total of 1846 samples for the transcriptomic analysis. Cohorts chosen included different geographic regions and multiple platforms; for instance, microarray studies were frequently generated on Affymetrix platforms, such as GPL570 and GPL96, whereas RNA sequencing studies were mostly obtained from multiple Illumina-based platforms. Sample sizes varied widely, ranging from 3 pairs to 230 pairs of tumor-normal samples. This ensured that the study covered different technical and individual study settings. More details, including the accession IDs and individual sample counts for each dataset, are provided in Table 1.

**TABLE 1:** Accession IDs, Sample counts, and expression profiling platforms of the datasets used in this study.

S.no	GEO ID	Experiment type	No. of samples		Platform	References
			Tumor	Normal		
1	GSE285296	RNA-seq	3	3	GPL20795	Bao et al. (2025)
2	GSE208099	Microarray	16	16	GPL21185	Nagao et al. (2023)
3	GSE248612	RNA-seq	6	6	GPL24676	Ding et al. (2024)
4	GSE224056	RNA-seq	5	5	GPL24676	Yang et al. (2025)
5	GSE172032	RNA-seq	4	4	GPL20301	Wang et al. (2022a)
6	GSE193453	RNA-seq	3	3	GPL20301	Zhang et al. (2022b)
7	GSE192468	RNA-seq	6	6	GPL21290	Zhen-Hua et al. (2020)
8	GSE184336	RNA-seq	231	230	GPL11154	Lou et al. (2022b)
9	GSE179252	RNA-seq	38	38	GPL20301	Lou et al. (2022a)
10	GSE174237	RNA-seq	6	6	GPL16791	Yu et al. (2021)
11	GSE158662	Microarray	3	3	GPL22755	-
12	GSE142000	RNA-seq	7	6	GPL23227	Wang et al. (2021)
13	GSE118916	Microarray	15	15	GPL15207	Li et al. (2019)
14	GSE109476	Microarray	5	5	GPL24530	Zhang et al. (2019)
15	GSE122401	RNA-seq	80	80	GPL16791	Mun et al. (2019)
16	GSE122796	RNA-seq	3	3	GPL11154	-
17	GSE118897	Microarray	10	10	GPL16686	Yang et al. (2019)
18	GSE103236	Microarray	10	9	GPL4133	(Chivu Economescu et al., 2010)
19	GSE84787	Microarray	10	10	GPL7077	Liu et al. (2018)
20	GSE79973	Microarray	10	10	GPL570	He et al. (2016)
21	GSE65801	Microarray	32	32	GPL14550	Li et al. (2015)
22	GSE63288	RNA-seq	22	22	GPL13393	Chang et al. (2016)
23	GSE63089	Microarray	45	45	GPL5175	Zhang et al. (2015)
24	GSE31811	Microarray	21	17	GPL6480	Kitamura et al. (2017)
25	GSE30727	Microarray	30	30	GPL5188	-
26	GSE56807	Microarray	5	5	GPL5175	Wang et al. (2014)
27	GSE29272	Microarray	134	134	GPL96	Wang et al. (2013)
28	GSE29998	Microarray	50	49	GPL6947	Holbrook et al. (2011)
29	GSE33335	Microarray	25	25	GPL5175	Cheng et al. (2012)
30	GSE27342	Microarray	80	80	GPL5175	Cui et al. (2011)
31	GSE19826	Microarray	12	12	GPL570	Wang et al. (2012)

To validate the differential expression of candidate genes, four independent external cohorts were utilized that covered distinct clinical populations: TCGA-STAD, GSE54129, GSE13911, and GSE66229. The prognostic relevance of selected genes was evaluated across three independent cohorts with survival data: TCGA-STAD, GSE62254, and GSE15459.

### 3.2 DEG analysis and RRA meta-analysis

Differential expression testing was conducted using appropriate methods across all 31 datasets after proper preprocessing based on their data types. Data normalization in RNA sequencing studies generally included VST or log2 transformations of FPKM/CPM (Fragments per kilobase of transcript per million mapped reads/counts per million) values. Microarray data were frequently normalized using quantile normalization and robust multiarray average (RMA). Before differential expression (DE) analysis, outliers were searched for, and two datasets (GSE84787 and GSE79973) underwent outlier removal. Across datasets, Limma and DeSeq2 were primarily used for DE analysis, and the results exhibited considerable heterogeneity and size variations. Table 2 provides the preprocessing steps, testing methods, and resulting gene counts of each dataset.

**TABLE 2:** Preprocessing steps, testing methods, and resulting gene counts from all 31 datasets.

S.no	GEO ID	Data type	Preprocessing method	DEG method	Number of DEGs identified
1	GSE285296	RPKM values	Log2 transformation	Limma	20,409
2	GSE208099	Raw data	Quantile normalization	Limma	62,976
3	GSE248612	NCBI-generated raw data for 11 samples	VST normalization	DESeq2 for initial analysis and Limma-voom for meta-DE compatibility	39,376
4	GSE224056	NCBI-generated raw counts	VST normalization	Only paired-end samples were considered; DESeq2 for initial analysis and Limma-voom for meta-DE compatibility	39,376
5	GSE172032	RPKM values	Log2 transformation	Limma	53,110
6	GSE193453	Raw count data	VST normalization	circRNA sequencing samples were not considered; DESeq2 for initial analysis and Limma-voom for meta-DE compatibility	10,257
7	GSE192468	FPKM values	Log2 transformation	Limma	19,273
8	GSE184336	Raw count data	VST normalization	DESeq2 for initial analysis and Limma-voom for meta-DE compatibility	18,386
9	GSE179252	Raw count data	VST normalization	DESeq2 for initial analysis and Limma-voom for meta-DE compatibility	18,177
10	GSE174237	Raw mRNA count data	TMM normalization	Limma-voom	47,303
11	GSE158662	Raw data	Quantile normalization	Limma	180,786
12	GSE142000	FPKM values	Log_2_ (+1) transformation	Limma	21,688
13	GSE118916	Raw intensity data	RMA normalization	Limma-voom	49,495
14	GSE109476	Raw data	Quantile normalization	Limma	62,738
15	GSE122401	Quantile normalized count data	Log2 transformation	Limma	34,908
16	GSE122796	Expression profiles of all genes	Extracted DE statistics	NA	20,345
17	GSE118897	Raw intensity data	RMA normalization	Limma	30,774
18	GSE103236	Raw data	Quantile normalization	Limma	45,015
19	GSE84787	Raw data	Quantile normalization; 2 outliers removed	Limma	62,975
20	GSE79973	Raw intensity data	RMA normalization; 2 outliers removed	Limma	28,053
21	GSE65801	Raw data	Quantile normalization	Limma	22,089
22	GSE63288	CPM data	Log2 Transformation	Limma	11,611
23	GSE63089	Raw intensity data	RMA normalization	Limma	17,260
24	GSE31811	Raw data	Quantile normalization	Limma	19,595
25	GSE30727	Raw intensity data	RMA normalization	Limma	17,260
26	GSE56807	Raw intensity data	RMA normalization	Limma	17,324
27	GSE29272	Raw intensity data	RMA normalization	Limma	13,515
28	GSE29998	Raw data	Neqc- background correction followed by quantile normalization	Limma	19,479
29	GSE33335	Raw intensity data	RMA normalization	Limma	17,324
30	GSE27342	Raw intensity data	RMA normalization	Limma	22,011
31	GSE19826	Raw intensity data	RMA normalization	Limma	54,975

The RobustRankAggreg algorithm (RRA) was used to integrate results across studies by ranking genes by adjusted p-value and then by log2 fold change (log2FC) in each dataset, and then combining the 31 ranked gene lists. Then, a score (RRA score) was assigned to all the genes, followed by Bonferroni correction to control multiple testing, where α was 0.05, m was 20,000 genes, producing a Bonferroni-adjusted threshold of 2.5 × 10^−6^. Following the RRA meta-analysis and the Bonferroni correction, 254 genes with consistent differential expression across both RNA-seq and microarray datasets were identified with significance. The uncorrected RRA scores of all the genes are provided in Supplementary Table 1A, and the list of Bonferroni-significant 254 genes and their scores is provided in Supplementary Table 1B.

### 3.3 WGCNA analysis and integration of RRA and WGCNA results

A total of 19 datasets met the criterion of a minimum sample size of 15 samples; each of these datasets was preprocessed and analyzed using the WGCNA R package. The pickSoftThreshold function was used to determine the optimal soft threshold power for each dataset. Signed networks were then constructed, modules were identified, and highly similar modules were merged. To identify tumor-correlated modules, module eigengenes (MEs) were correlated with the tumor-versus-normal trait, and genes with GS > 0.2 and MM > 0.8 were selected as hub genes from these modules. Table 3 summarizes the preprocessing methods and soft-threshold powers used, along with the number of hub genes from tumor-correlated modules in each dataset. A presence-absence matrix was constructed using the gene symbols of these hub genes to calculate their frequency of occurrence across datasets, and the genes were then categorized into three tiers: high confidence, medium-confidence and exploratory. The sizes of these three categories were defined at three levels through pre-processing:

i. Original sizes based on the presence-absence matrix: High/Medium/Exploratory- 655/3390/3273 (Supplementary Tables2A-C provides the dataset details and the frequencies of the genes from all three categories)
ii. Unique gene symbols after mapping to HGNC symbols, and removal of duplicates: High/Medium/Exploratory- 653/3357/3252
iii. Gene symbols after restricting to the common background universe (|U| = 27,740): High/Medium/Exploratory- 653/3355/3229

**TABLE 3:** Preprocessing methods, soft threshold powers used, and the number of hub genes from tumor-correlated modules from all 19 datasets.

s.no	Dataset	Preprocessing method	Soft-threshold power	Hub genes tumor correlated modules
1	GSE208099	Quantile normalization	8	4034
2	GSE184336	Filtered low-count genes and VST normalization	7	1441
3	GSE179252	Filtered low-count genes and VST normalization	5	2009
4	GSE118916	RMA normalization, Background correction and log2 transformation	18	4298
5	GSE122401	Pre-normalized (quantile normalized)	5	1378
6	GSE118897	RMA normalization	6	1837
7	GSE103236	Quantile normalization	5	4733
8	GSE84787	Quantile normalization	4	3862
9	GSE79973	RMA normalization	10	4463
10	GSE65801	Quantile normalization	6	3678
11	GSE63288	Pre-normalized data (TMM normalization from edgeR)	6	1831
12	GSE63089	RMA normalization	4	1277
13	GSE31811	Quantile normalization	14	21
14	GSE30727	RMA normalization	6	2140
15	GSE29272	RMA normalization	6	1498
16	GSE29998	Log2 transformation followed by quantile normalization	6	264
17	GSE33335	RMA normalization	7	2332
18	GSE27342	RMA normalization	6	1056
19	GSE189826	RMA normalization	12	3276

To determine whether the overlaps were different from what would be expected by chance, one-sided Fisher tests were conducted to compare each WGCNA category with the RRA gene set, and the summarized effect sizes, along with OR and RF, were recorded. Of the 653 genes from the high-confidence WGCNA category, 142 overlapped with the RRA list with an OR of 66.80, RF of 23.75, p-value of 1.75 × 10 (^−165^), and FDR of 5.25 × 10 (^−165^), thus exhibiting significant enrichment. The 3355 genes from the medium-confidence WGCNA category exhibited a significantly enriched overlap of 94 genes with the RRA set with an OR of 4.36, RF of 3.06, p-value of 1.08 × 10 (^−24^), and FDR of 1.6 × 10 (^−24^). In contrast, among the 3,229 genes from the exploratory subset of WGCNA, only 10 genes overlapped with the RRA set, with an OR of 0.31, an RF of 0.34, a p-value, and an FDR approximately equal to 1. The contingency counts and other statistical values, including the a, b, c, and d values for each category and the lower bounds of the CI for the odds ratio, are provided in Supplementary Table 2D. The results of Fisher’s test demonstrated that the exploratory category did not exhibit significant enrichment, whereas the overlap between the high-confidence and medium categories was statistically significant. Thus, a total of 236 genes (142 from high-confidence overlap and 94 from medium-confidence overlap) were selected for further analysis, and Supplementary Table 2E lists these genes along with their WGCNA category and RRA scores.

### 3.4 PPI analysis and hub gene selection

**FIGURE 2:**
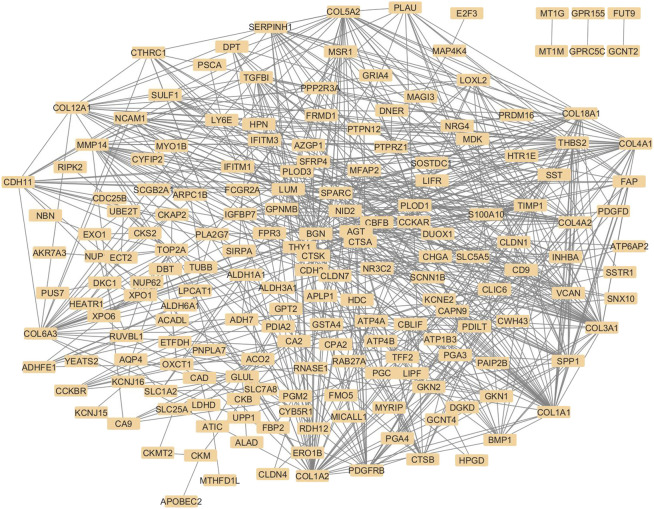
Protein-protein interaction network diagram with 236 nodes, where each node is a protein. Grey lines between the nodes represent their STRING interactions. The nodes are densely connected, and proteins like collagen family members, including COL1A1, COL1A2, COL5A2, COL 18A1 are among the major visible nodes with many edges. Weakly connected nodes like FUT9, MT1G, and GPRC5C appear towards the periphery of the diagram. The overall pattern of the diagram suggests a strong interconnection between the nodes.

The STRING database was employed to retrieve protein-protein interactions for the 236 overlapping genes, considering a minimum interaction score of 0.4. As STRING lacked entries for Aquaporin-4 (AQP4), its interactions were supplemented using the STITCH database, and the combined network was then imported into Cytoscape. Figure 2 shows the combined PPI network of the 236 hub genes.

**FIGURE 3:**
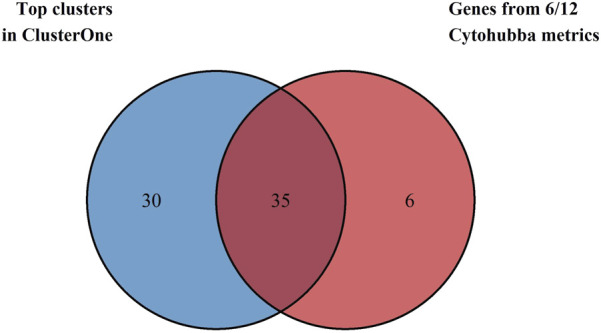
Venn diagram depicting the intersection between 41 Cytohubba-derived hub genes (blue) and 65 proteins from ClusterONE modules (red). Thirty-five genes were common to both methods and were selected as candidate genes for further analysis.

All twelve metrics of the Cytohubba plugin were applied to the network to analyze and rank the topology of the nodes, and the top 40 genes identified by each measure are depicted in Supplementary Figures 1A-L. The 41 genes identified by at least six of the twelve metrics (≥50% consensus) were selected as Cytohubba-derived hub genes, and their details are provided in Supplementary Table 3A. Simultaneously, the same network was used to identify densely connected protein complexes using the ClusterONE web. Ten significant clusters were identified with a minimum density of 0.3 and p-values <0.05, as shown in Supplementary Table 3B, and were then prioritized based on a minimum size of 7 genes. Four clusters (Clusters 1,2, 5, and 6) with 65 component genes were chosen as key clusters due to their high quality and low p-values. For robust selection of hub genes, the 35 genes that overlapped between Cytohubba hub genes and genes from key ClusterONE modules were selected for further analysis. Figure 3 shows the Venn diagram used to visualize the overlapping hub genes from the analyses above.

### 3.5 Functional enrichment analyses

**FIGURE 4:**
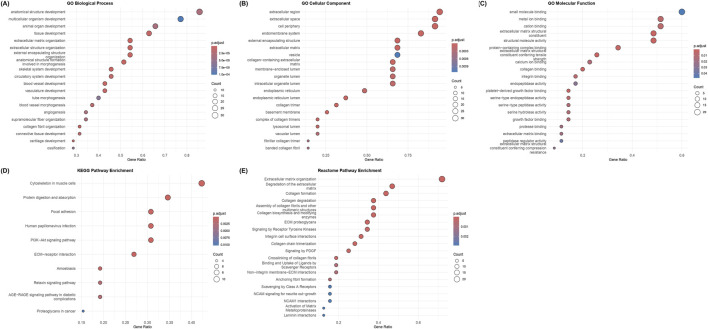
GO, KEGG, and Reactome enrichment analyses of the 35 chosen hub genes. Color indicates the adjusted p-value, and dot sizes are proportional to gene counts: **(A)** enriched GO Biological process (BP) terms, **(B)** enriched GO cellular component (CC) terms, **(C)** enriched GO molecular function (MF) terms, **(D)** enriched KEGG pathways, and **(E)** enriched Reactome pathways. Terms related to ECM organization, functions, and interactions are enriched in all five categories.

To understand the biological roles of the 35 hub genes, GO (BP, CC, and MF), KEGG, and Reactome pathway enrichment analyses were conducted. In the GO-BP enrichment, genes involved in extracellular matrix (ECM) organization, cell adhesion, angiogenesis, blood vessel morphogenesis, cell migration and other related processes in tissue remodeling and tumor progression were significantly enriched. Some of the recurrently enriched genes include multiple collagen family members and other genes such as MMP14, TGFBI, SERPINH1, THY1, FAP, SULF1, PDGFRB, SPARC, NID2, LOXL2, and THBS2. Figure 4A shows the dot plot representation of the top enriched GO biological process terms.

The cellular localization of the enriched genes was primarily in the extracellular matrix category, along with collagen-containing ECM, extracellular space, basement membrane, endoplasmic reticulum lumen, and collagen trimers. The key genes enriched in this category include VCAN, MMP14, SERPINH1, BGN, SPARC, TGFB1, CTSB, LOXL2, NID2 and collagen-family genes such as COL1A1, COL3A1, COL4A2, COL5A2, COL4A2 and COL18A1. Figure 4B displays the dot plot representing the enriched GO cellular component terms.

The molecular functions (MF) category mainly consisted of structural constituents of the ECM and binding activities to collagen and growth factors, both of which are related to matrix interactions and cell signaling. The key genes recurring in the top GO-MF terms were the collagen and ECM proteins like COL6A3, COL1A1, COL1A2, COL4A1, COL3A1, SPARC, NID2, and VCAN, contributing to the structural component function and genes like FAP, SERPINH1, PDGFBR, MMP14, LUM and CTSK contributing to growth factor and collagen binding functions. Figure 4C shows the dot plot of the top GO Molecular Function terms that were enriched in the analysis.

The KEGG pathway analyses revealed key pathways in cell-matrix interactions and cancer signaling, including the PI3K-Akt pathway, focal adhesion, ECM-receptor interactions, and protein digestion and absorption. Collagen family members like COL6A3, COL4A1, COL4A2, COL1A2, and COL1A1, along with genes such as THBS2, PDGFRB, LUM, and SPP1, were among the key genes in KEGG-enriched pathways. Figure 4D depicts the dot plot of the key pathways that were enriched in the KEGG analysis.

The Reactome pathway enrichment analysis revealed major pathways in the organization and degradation of ECM, the formation, assembly, biosynthesis, and degradation of collagen, and interactions, including integrin/non-integrin interactions and PDGF/RTK signaling pathways. Apart from major collagen genes such as COL1A2, COL1A1, COL3A1, COL4A2, COL5A2, COL18A1, and COL4A1, which are part of multiple pathways, other genes, such as BMP1, PLOD1, PDGFRB, SPP1, CTSK, and MMP14, were also members of the enriched ECM/growth factor pathways. Figure 4E displays the dot plot of the top Reactome pathways that were enriched.

The genes that were not enriched in any of the above analyses (p.adj ≤0.05) and the ones without at least 10 occurrences across the ECM/cancer-related significant terms and pathways were excluded from being considered as hub genes. Supplementary Tables 4A-E provides the results of the GO, KEGG and Reactome enrichment analyses. The final hub genes that were nominated as the final 29 potential therapeutic candidates were BMP1, COL1A2, LUM, SPP1, THBS2, VCAN, COL1A1, COL3A1, CTSK, MMP14, PDGFRB, SERPINH1, THY1, BGN, COL4A2, COL5A2, FAP, SPARC, TGFB1, COL12A1, COL18A1, COL4A1, CTSB, LOXL2, NID2, COL6A3, PLAU, PLOD1, and SULF1.

### 3.6 Validation of differential expression

**FIGURE 5:**
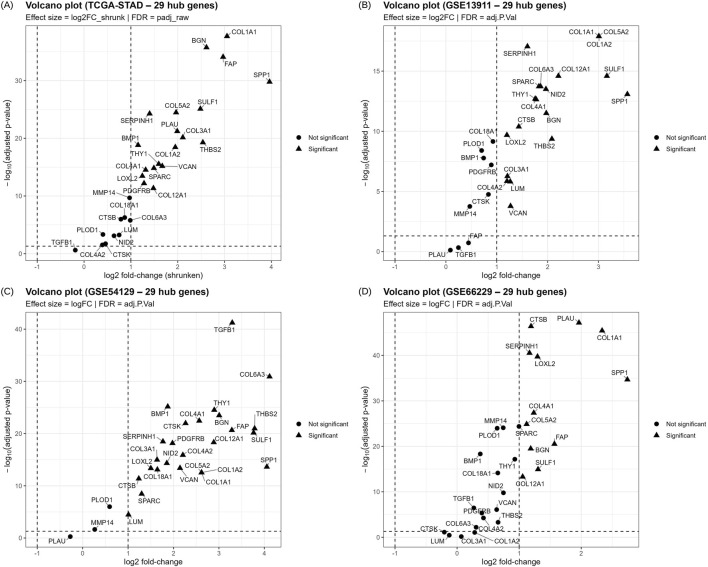
Volcano plots depicting the expression of 29 candidate therapeutic targets in 4 independent cohorts: **(A)** TCGA-STAD; **(B)** GSE13911; **(C)** GSE54129; and **(D)** GSE66229. X-axis is the log2FC and y-axis is the -log10 FDR; Each point represents a gene, where circles represent non-significant and triangles represent FDR-significant genes.

For validation of the differential expression of the 29 hub genes, four independent cohorts from distinct populations were used: RNA sequencing data from TCGA-STAD (412 tumors and 36 normal samples) and three microarray datasets from GPL570. GSE54129 included 111 tumors and 21 normal samples from China; GSE13911 included 38 tumors and 31 normal samples from Italy; and GSE66229 included 300 tumors and 100 normal samples from the Asian Cancer Research Group (ACRG). All 29 hub genes were detected in the validation cohorts and quantified. Most of the candidates satisfied the |log2FC| ≥ 1 and FDR ≤0.05 in individual cohorts, and their expression results are provided in Supplementary Tables 5A-D. The volcano plots of these genes from all four cohorts are given in Figures 5A–D. Regardless of the individual results, a meta-analysis of the 29 genes was conducted using the REML model; 22 genes were significant (BH-FDR ≤0.05) with 95% CI and showed consistent dysregulation across cohorts. ECM and stromal genes, such as COL1A1, BGN, SPP1, MMP14, and PLOD1, were consistently higher in tumors with positive pooled log2FC values. Heterogeneity (I^2^) values were often high because the study combined RNA sequencing and microarray data from distinct populations, yet the pooled effects showed consistent direction. LOO studies were conducted for sensitivity, and most genes were retained as they did not exhibit any sign flips or p-values ≥0.05, regardless of which cohort was removed. CTSK, PLAU and TGFB1 were excluded from downstream analyses due to insignificant REML results, and COL3A1, COL4A2, COL6A3, and LUM were excluded due to insignificant p values in the LOO analysis. Supplementary Table 5E displays the REML and LOO results of the 29 genes. The 22 genes out of the 29 candidates with validated differential expression include BGN, BMP1, COL12A1, COL18A1, COL1A1, COL1A2, COL4A1, COL5A2, CTSB, FAP, LOXL2, MMP14, NID2, PDGFRB, PLOD1, SERPINH1, SPARC, SPP1, SULF1, THBS2, THY1, and VCAN. Forest plots of these genes were generated with log2FC per study, with pooled REML at the bottom, and are provided in Supplementary Figures 2A-Z, 2a-c.

### 3.7 Survival analysis and final target selection

For analyzing the prognostic effects of the selected 22 genes, three independent cohorts were utilized: TCGA-STAD had 445 tumor samples with OS and covariates mentioned such as age (437/445), sex (445/445) and stage (77/445); GSE62254 had 300 tumors, GSE15459 had 192 tumors and covariates such as age, sex and stage were available for all the samples from both datasets. Gene expression for the 22 genes was standardized within each cohort, and KM plots were generated for visualization, with Q4 vs. Q1 for each gene. Supplementary figures 3A-V, 4A-V, and 5A-V show the KM plots of the 22 genes with the n-at-risk tables from all three cohorts.

Across all three cohorts, higher expression of the 22 genes was often associated with worse OS. In the univariate and multivariate Cox models adjusted for age and sex, TCGA data showed that many genes, including BGN, FAP, NID2, PDGFRB, THY1, VCAN and some collagen genes were significant with adjusted-HR >1 and FDR ≤0.05. Similarly, univariate and multivariate models adjusted for age, sex, and stage across both GSE62254 and GSE15459 showed independent associations between gene expression and adverse survival outcomes for many genes, including COL18A1, COL4A1, BGN, NID2, VCAN, and THBS2, among others. Supplementary Tables 6A-F provides the univariate and multivariate results of all three cohorts. The results from these cohorts indicated consistent associations between ECM-related gene dysregulation and poorer survival outcomes. The power of the interaction analyses among genes and stages in TCGA was restricted by the small number of cases with stage details (n = 77), and by the lack of significant interactions between any genes and stages with FDR ≤0.05. Within the GSE62254 cohort, some genes, like CTSB and SPP1, showed gene-level associations with multiple stages, but after BH correction, no genes had significant interactions. For the GSE15459 cohort, no interactions were significant after BH correction within genes or across genes. Thus, there was no consistent evidence that the pathological stages could change the prognostic significance of any of the genes.

REML analysis of the univariate results across all cohorts identified 17 of the 22 validated genes as consistent markers (FDR ≤0.05) whose higher expression was associated with worse OS. The percentage of variation across cohorts (I^2^) was low to moderate for most of these genes, and in the LOO analysis, there was no loss of significance or sign changes. Even though they exhibited consistent tumor-normal dysregulation, five genes (COL12A1, CTSB, PLOD1, SPP1, and SULF1) were excluded from the final candidate list as they did not satisfy the meta-analysis criteria and exhibited loss of prognostic significance. This discordance suggests that differential expression does not necessarily mean the gene would contribute to the prognostic significance. Supplementary Tables 6G-H provides the results of the REML and LOO analyses. Forest plots of all the 22 genes displaying the cohort-specific HRs with 95% CIs, pooled HRs, and other statistical values like I^2^, and c-indexes are provided as Supplementary Figures 6A-F. The significant genes in the survival analysis were shown to be key players in ECM remodeling with stromal significance, including collagen genes, matrix-processing genes, and cancer-associated fibroblasts (CAFs) or stromal markers. Thus, the 17 final potential therapeutic candidates with significant prognostic associations (the pooled HR > 1, pooled FDR ≤0.05, HR > 1 in ≥2/3 cohorts with no sign changes in LOO) are BGN, BMP1, COL18A1, COL1A1, COL1A2, COL4A1, COL5A2, FAP, LOXL2, MMP14, NID2, PDGFRB, SERPINH1, SPARC, THBS2, THY1, and VCAN.

### 3.8 ADMET profiling and ligand preparation

**FIGURE 6:**
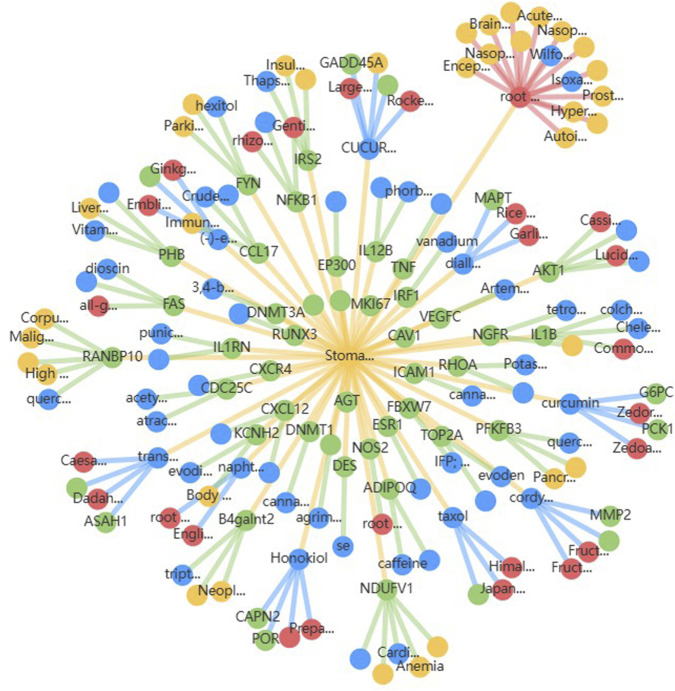
Compound-target interaction network for 58 stomach neoplasm-related phytochemicals retrieved from TCMBank, where blue represents the candidate compounds and green represents the potential targets. Red nodes represent the herbs in which these compounds are present, and yellow nodes represent other diseases where the candidate compounds may have potential roles.

For the 17 chosen targets, a systematic literature search was conducted to identify prior *in silico*, *in vitro*, or animal model-based experiments targeting them in gastric cancer. It was found that there is limited data on reports of natural compound or drug testing for 4 of the selected genes in gastric cancer. As a result, those genes were retained for docking analysis, and the other 13 genes, which had been previously targeted, were excluded. The source of phytochemicals was TCMBank, where 58 compounds were retrieved for the search term ‘stomach neoplasm’. Figure 6 shows the interaction diagram of the 58 compounds obtained from TCMBank. SMILES strings for the 58 compounds were utilized for ADMET evaluation, and SwissADME was used for ADME evaluation to prioritize compounds that obeyed Lipinski’s rule of five, exhibited high gastrointestinal (GI) permeability, and showed favorable bioavailability scores. ProTox-III was used for toxicity profiling to retain compounds in toxicity classes IV and V (low acute toxicity). Only 14 compounds exhibited acceptable ADMET profiles, and their 3D structures were retrieved in SDF format from PubChem for docking. Supplementary Table 7 presents the ADMET profiles of the selected 14 compounds. The reference compound chosen was 5-fluorouracil (5-FU), a common small-molecule chemotherapeutic agent in the treatment of gastric cancer. These compounds were energy-minimized and converted to formats suitable for docking (PDBQT format) using OpenBabel.

### 3.9 Target structure selection and preparation

After the targeted literature search, it was shown that most of the final therapeutic candidates had already been studied as targets in in silico, *in vitro*, or *in vivo* settings, and four targets (THBS2, VCAN, THY1, and NID2) had limited information on their use as targets. For THBS2, PDB entry 1YO8 containing the C-terminal domain was used as it was the highest-quality entry available in the RCSB PDB. This PDB model was directly cleaned and protonated using MGL tools. As experimental structures were not available, AlphaFold models corresponding to the Uniprot entries were used for VCAN (D6RGZ6), THY1 (P04216), and NID2 (Q14112). For these models, residues with confidence scores less than 50 (pLDDT<50) were removed using PyMOL, and missing loops were remodeled using SWISS-MODEL. Additionally, steric clashes were repaired using PRAS for NID2, and the energy minimization was performed in SwissPDBViewer for the THY1 model (Nnyigide et al., 2022; Guex and Peitsch, 1997). The primary binding pocket information of all the finalized structures was obtained using PrankWeb. Finally, after protein preparation, all structures were saved in PDBQT format for docking.

### 3.10 Molecular docking and lead compound selection

**FIGURE 7:**
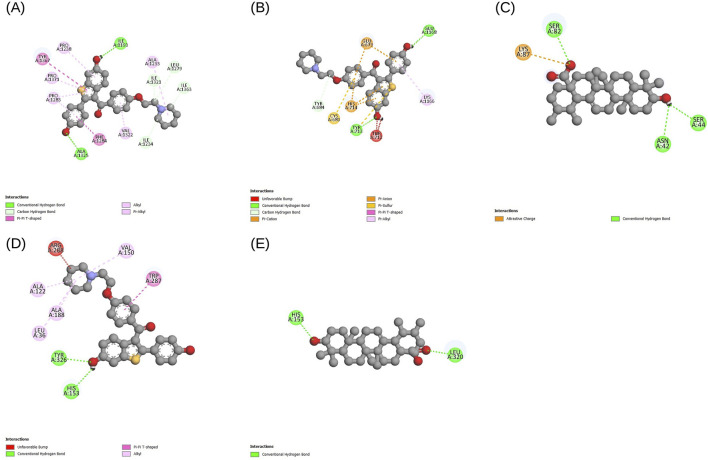
Docking interaction diagrams for the 4 final targets with their top-scoring ligands, visualized in Discovery Studio. Each panel shows the ligand bound in the protein’s primary pocket, defined using Prankweb: **(A)** NID2-Evoden; **(B)** THBS2-Evoden; **(C)** THY1-Ursolic acid; **(D)** VCAN-Evoden; **(E)** VCAN-Ursolic acid. All lead compounds achieved more binding affinities than the reference molecule 5-FU.

AutoDock Vina was used for docking simulations against each protein’s primary binding pocket. Additionally, 5-FU was docked to these sites as a reference compound, and each ligand-target pair was run in triplicate with different random seeds. The best affinities were consistent across the runs (variation less than 1 kcal mol^-1^), and lead phytocompounds were chosen based on the lowest binding affinities. All selected compounds exhibited better binding scores than the reference drug 5-FU, and the phytocompound showed the highest affinity for VCAN, NID2, and THBS2. For VCAN, along with evoden, ursolic acid scored the best affinity of −9.5 kcal mol^-1^ in all triplicates. Evoden showed high affinities of −10.4, −10.4, −10.3 kcal mol^-1^ with NID2 and a binding affinity of −9.5 kcal mol^-1^ with THBS2 across all the runs. Ursolic acid scored best with the THY1 model with −6.9 kcal mol^-1^ across the runs, even though it is higher than the reference drug (−4.4 kcal mol^-1^); it is comparatively low, probably due to the shallow ectodomain of the protein. Table 4 summarizes the docking and interaction results with binding affinities (triplicate mean ± standard deviation, SD) of the four targets. The interactions between the top-scoring ligand model and targets were visualized using Discovery Studio Visualizer, and the interactions were noted. Figures 7A–E depicts the interaction diagrams of all the target-ligand complexes.

**TABLE 4:** Structure and grid information, binding affinities and interaction results of all 4 targets and their top-scoring ligands.

Target	Structure used	Grid center (x,y,z)	Binding affinity to reference drug	Compound	Binding affinity (Kcal/mol)	Interactions
NID2	Q14112 (AlphaFold)	(3.0147, 10.3555,32.6509)	−5.5 ± 0.0	Evoden	−10.37 ± 0.06	ILE A:1321
						LEU A:1279
						ILE A:1363
						PRO A:1238
						PRO A: 1371
						PRO A: 1283
						VAL A: 1322
						ALA A: 1233
						PHE A: 1284
						TYR A:1367
						ALA A:1325
						ILE A:1193
THBS2	IYO8 (PDB)	(19.9387, 7.2468,44.3941)	−5.3 ± 0.0	Evoden	−9.5 ± 0.0	TYR A:684
						GLU A:679
						HIS A:714
						CYS A:680
						TYR A:713
						GLU A: 1168
						TYR A:713
						LYS A 1166
						THR A:712
THY1	P04216 (AlphaFold)	(4.6729, 11.3848, −6.0522)	−4.4 ± 0.0	Ursolic acid	−6.9 ± 0.0	SER A: 82
						SER A:44
						ASN A:42
						LYS A:87
VCAN	D6RGZ6 (AlphaFold)	(7.4391, 1.8036, −2.3163	−5.07 ± 0.6 −5.07 ± 0.6	Evoden Ursolic acid	−9.5 ± 0.0 −9.5 ± 0.0	HIS A:153
						TYR A:326
						VAL A: 150
						ALA A: 122
						ALA A: 188
						LEU A:36
						TRP A:287
						ARG A: 288
						HIS A:153
						LEU A:320

Conventional  Hydrogen  Bond .

Pi-Pi  Stacked/T-shaped .

Alkyl/ Pi-Alkyl .

Pi- Anion/Cation .

Pi-Sulfur .

Attractive  Charge .

Unfavorable  Bump .

Carbon-hydrogen  bond .

Across these targets, the docked complexes exhibited non-classical interactions, including alkyl, pi-alkyl, and carbon-hydrogen bonds in addition to the standard donor-acceptor hydrogen bonds. Unfavorable bumps may be due to shallow geometry, imperfections in the modeled regions, or the absence of cofactors and metal ions, suggesting areas for further model refinement and experimental validation. However, triplicate runs produced reproducible results across the targets, where ursolic acid and evoden showed good binding affinities to the chosen targets.

## 4 Discussion

This study utilized an integrative pipeline to finalize seventeen potential therapeutic targets with prognostically adverse effects in gastric cancer: BGN, BMP1, COL18A1, COL1A1, COL1A2, COL4A1, COL5A2, FAP, LOXL2, MMP14, NID2, PDGFRB, SERPINH1, SPARC, THBS2, THY1, and VCAN. It combined the Bonferroni-corrected RRA genes with hub genes from WGCNA modules, followed by network analysis combining topological and clustering analyses, expression validation, and survival meta-analysis to narrow down to 17 potential therapeutic targets with druggable roles in GC. Functionally, all final genes were either part of stromal/ECM or tumor-microenvironment regulation, and the presence of collagen pathways, matrix proteolysis and cross-linking, along with fibroblast signaling, provided further evidence that ECM remodeling promotes GC tumor as well as immune invasion and therapy resistance (Moreira et al., 2020).

The first category identified had basement membrane-related functions and included multiple collagen genes, including COL1A1, COL4A1, COL1A2, COL5A2, and COL18A1, along with the linker NID2. The collagen genes are often upregulated in GC; collagen type Ⅰ alpha 1 chain (COL1A1) and collagen type Ⅰ alpha 2 chain (COL1A2) upregulation are shown to be associated with poor survival, and collagen type Ⅳ alpha 1 chain (COL4A1) is shown to promote the progression of GC and multidrug resistance. (Li J. et al., 2016; Qian et al., 2025). Enhanced migration and a worse prognosis are often associated with collagen type Ⅴ alpha 2 chain (COL5A2) overexpression, and Nidogen 2 (NID2) is shown to remodel the basement membrane to facilitate metastasis. (Yu et al., 2019; Tan et al., 2021). Meanwhile, high collagen type XVIII alpha 1 chain (COL18A1) expression and higher serum levels of endostatin encoded by this gene are suggested to be correlated with metastasis and unfavorable prognosis. This gene has also been tested as an anti-angiogenic target, along with chemotherapy, using recombinant human endostatin (Endostar) due to its ability to inhibit angiogenesis (Lee et al., 2010; Zhou et al., 2011; Wang et al., 2015). The second category, represented by the selected genes, is collagen processing, with components such as BMP1, SERPINH1, MMP14, and LOXL2 that enable ECM invasion. It is demonstrated that bone morphogenetic protein 1 (BMP1) overexpression is common in GC, and when suppressed, it inhibits tumor cell motility (Hsieh et al., 2018). Serpin family H member 1 (SERPINH1) enables collagen maturation, and thus its knockdown is shown to reduce the proliferation, invasion, and migration of GC cells (Tian et al., 2020). Other selected members of this category are matrix metallopeptidase 14 (MMP14), whose serum levels are often associated with adverse survival outcomes in GC, and lysyl oxidase-like 2 (LOXL2), which mediates the progression of GC (Kasashima et al., 2014; Kasurinen et al., 2018). Additionally, a TME-related category involving growth factor and CAF activity was identified, with genes such as FAP and PDGFRB; high platelet-derived growth factor receptor beta (PDGFRB) is associated with reduced survival outcomes when receiving S-1 adjuvant chemotherapy. (Higuchi et al., 2017). Meanwhile, fibroblast activation protein-positive (FAP+) CAFs are shown to promote GC cell growth and confer resistance to immune checkpoint inhibitors in xenograft models. (Wen et al., 2017). Another important category of selected genes is the modulators that enable cross-talk between cells and the matrix: VCAN, SPARC, THBS2, BGN, and THY1. Secreted protein acidic and cysteine-rich (SPARC) overexpression is correlated with poorer OS in GC patients. Meanwhile, thrombospondin-2 (THBS2) has been shown to promote stemness and the progression of GC through activation of the Notch pathway. It was found that THBS2 can act as a prognostic and diagnostic marker due to its roles in ECM regulation and angiogenesis (Li Z. et al., 2016; 2021; Chang et al., 2024). The dysregulation of versican (VCAN) leads to tumor invasion. It has a significant correlation with worsened outcomes; its upregulation in gastric cancer is shown to promote cancer cell proliferation and is associated with enhanced tumor invasion and HER2 positivity (Li et al., 2020). Recent studies also suggested the combined effect of VCAN and THBS2 to accelerate the growth of gastric cancer (Wang et al., 2023). Additionally, biglycan (BGN) upregulation has shown potential association with immune infiltration and worse survival outcomes in GC (Zhang S. et al., 2022). Another therapeutic target identified is the glycoprotein Thymus cell antigen 1 (THY1), also known as CD90, which is suggested to be a potential immunotherapy target that could be used for assessing the TME status (Hu et al., 2020). High expression of THY1 has also demonstrated evidence of modulating SPARC expression and thereby, inhibition of apoptosis of GC cells (ZHU et al., 2015).

The 17 identified targets are central to the druggable aspects of the tumor microenvironment; anti-PDGFR approaches, LOXL2 inhibitors, MMP14 targeting, and FAP are well-studied strategies in GC treatment. ECM components such as VCAN, THBS2, SPARC, BGN and collagen modulators are generally easily targetable by small molecules. A literature survey confirms that there is limited information on studies testing natural compounds or small-molecule drugs against four of these targets, thereby leaving a therapeutic targeting gap that this study aimed to address. The molecular docking analyses conducted identified evoden and ursolic acid as promising lead compounds against these targets. However, the study was limited by the restricted scope of compounds, which involved a single natural compound library. Thus, the lead compounds were suggested as potential therapeutic leads that need extensive testing in the future. Another limitation was the target models, which lacked bound cofactors and ions, which may have contributed to weak interactions; thus, they require further structural modifications to display their potential interactions accurately. Furthermore, the study did not include single-cell datasets that would have reflected the localized expression of the hub genes. The study recommends expanding chemical screening to include diverse natural product databases and drug libraries, as well as molecular dynamics simulations, and to include crucial *in vitro* and *in vivo* validations in the future. It also recommends including analyses of single-cell GC data to confirm the stromal and pericellular localization of the hub genes to ensure effective targeting of GC cells. To conclude, this study identified 17 targets that could lead to more effective, personalized therapeutic interventions. However, as the study is entirely computational, the expression patterns and prognostic significance of the hub genes can be confirmed only through future experimental validation, including qPCR/Western blotting and immunohistochemistry analyses of tissue samples.

## Data Availability

The original contributions presented in the study are included in the article/Supplementary Material, further inquiries can be directed to the corresponding author.
