# Supplementary material for: Identification and validation of tumor microenvironment-related therapeutic targets in gastric cancer using integrated multi-omics and molecular docking approaches
Source: Front Bioinform. 2025 Dec 10;5:1654326. doi: 10.3389/fbinf.2025.1654326 (PMC12727970; doi:10.3389/fbinf.2025.1654326)
Supplement: Supplementary file 6 [file DataSheet2.docx]

Supplementary FIGURES_3,4,5&6

Identification And Validation of Tumor Microenvironment-Related Therapeutic Targets in Gastric Cancer Using Integrated Multiomics and Molecular Docking Approaches

1. **Supplementary figure 3**


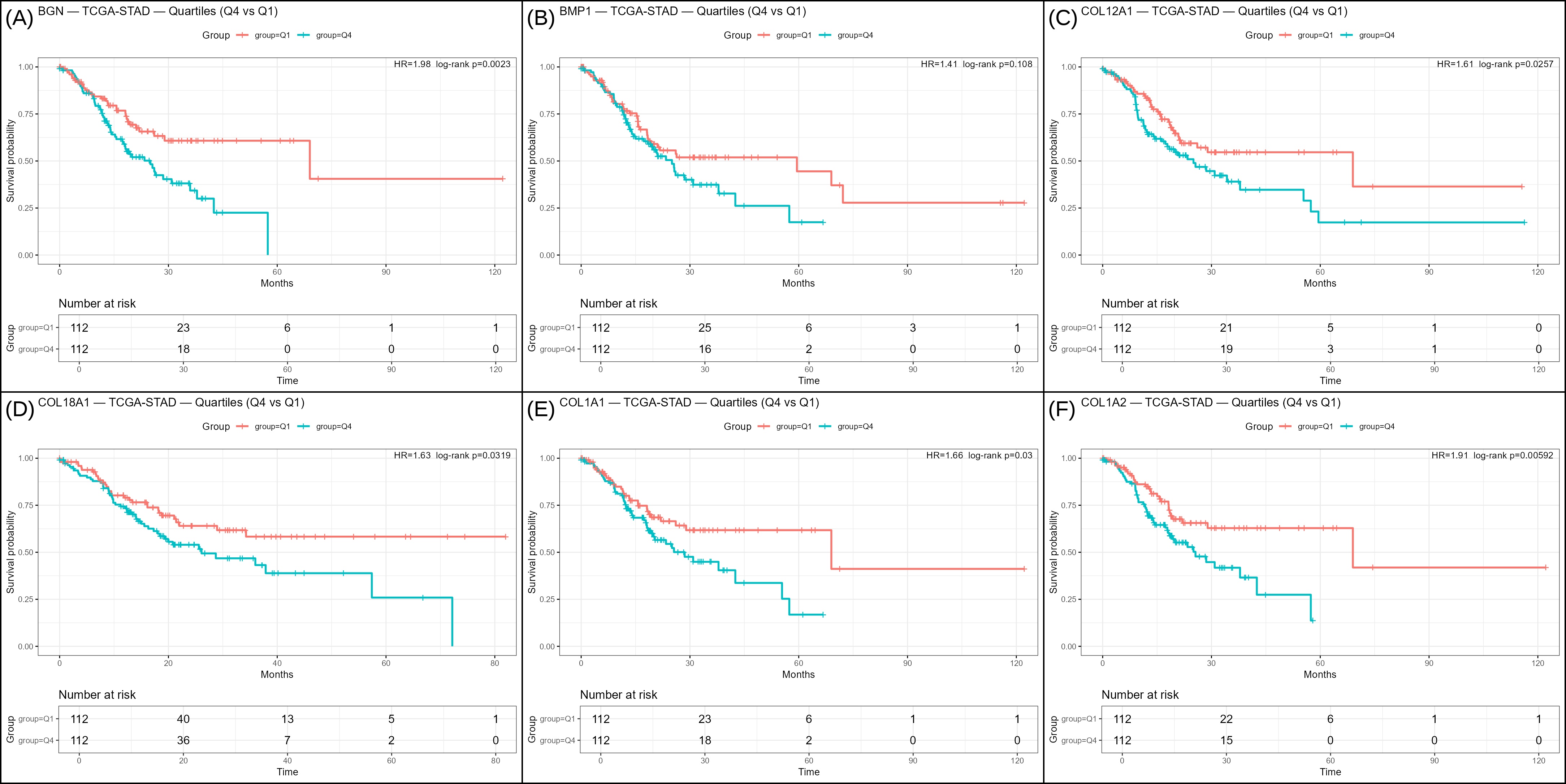


**Supplementary figure 3A-F**- KM plots using the top 25% vs the bottom 25% of expression (Q4 vs Q1) within the TCGA cohorts for BGN, BMP1, COL12A1, COL18A1, COL1A1, and COL1A2 with log-rank p-values, and n-at-risk tables


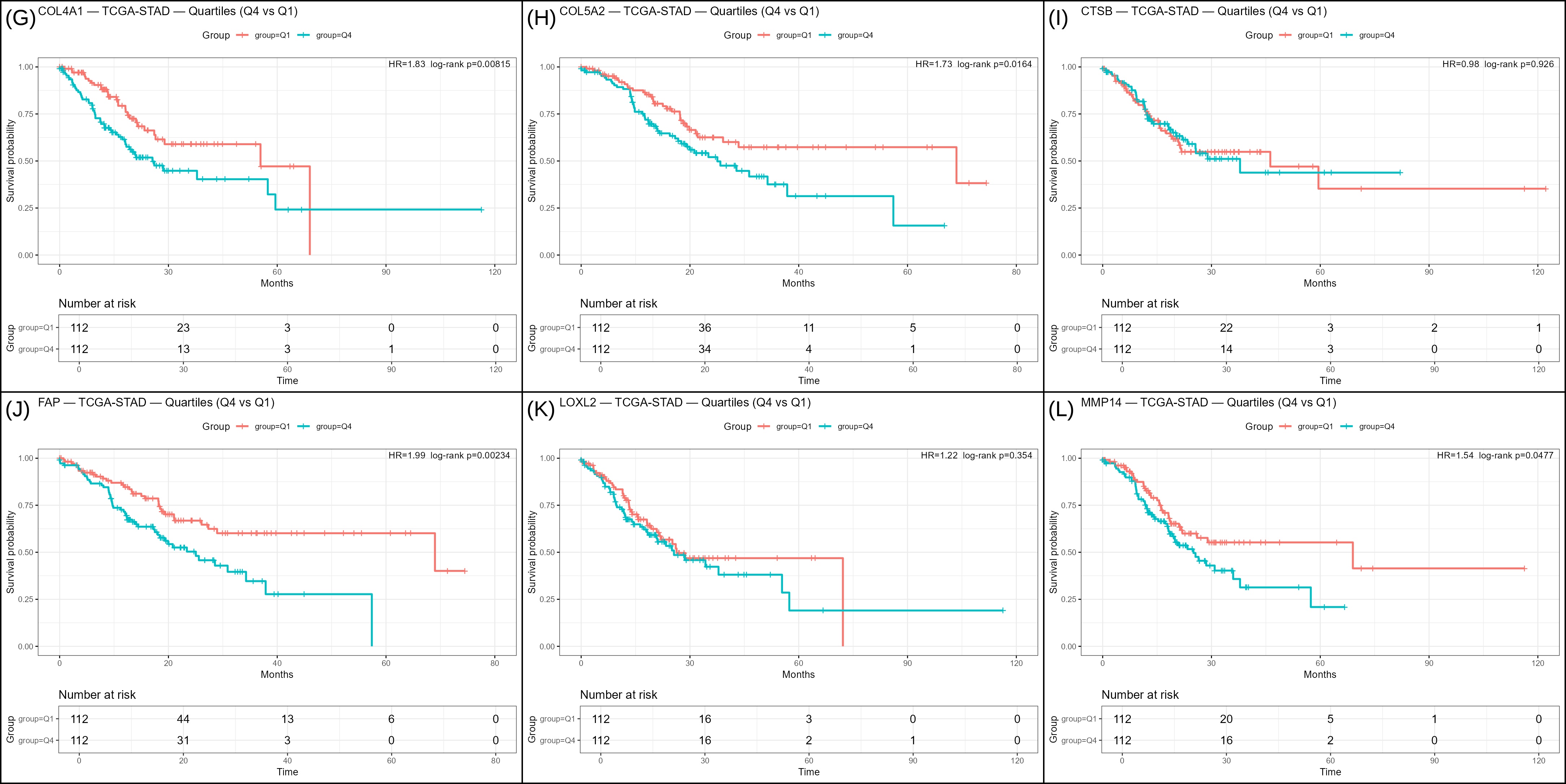


**Supplementary figure 3G-L**- KM plots using the top 25% vs the bottom 25% of expression (Q4 vs Q1) within the TCGA cohort for COL4A1, COL5A2, CTSB, FAP, LOXL2, and MMP14 with log-rank p-values, and n-at-risk tables


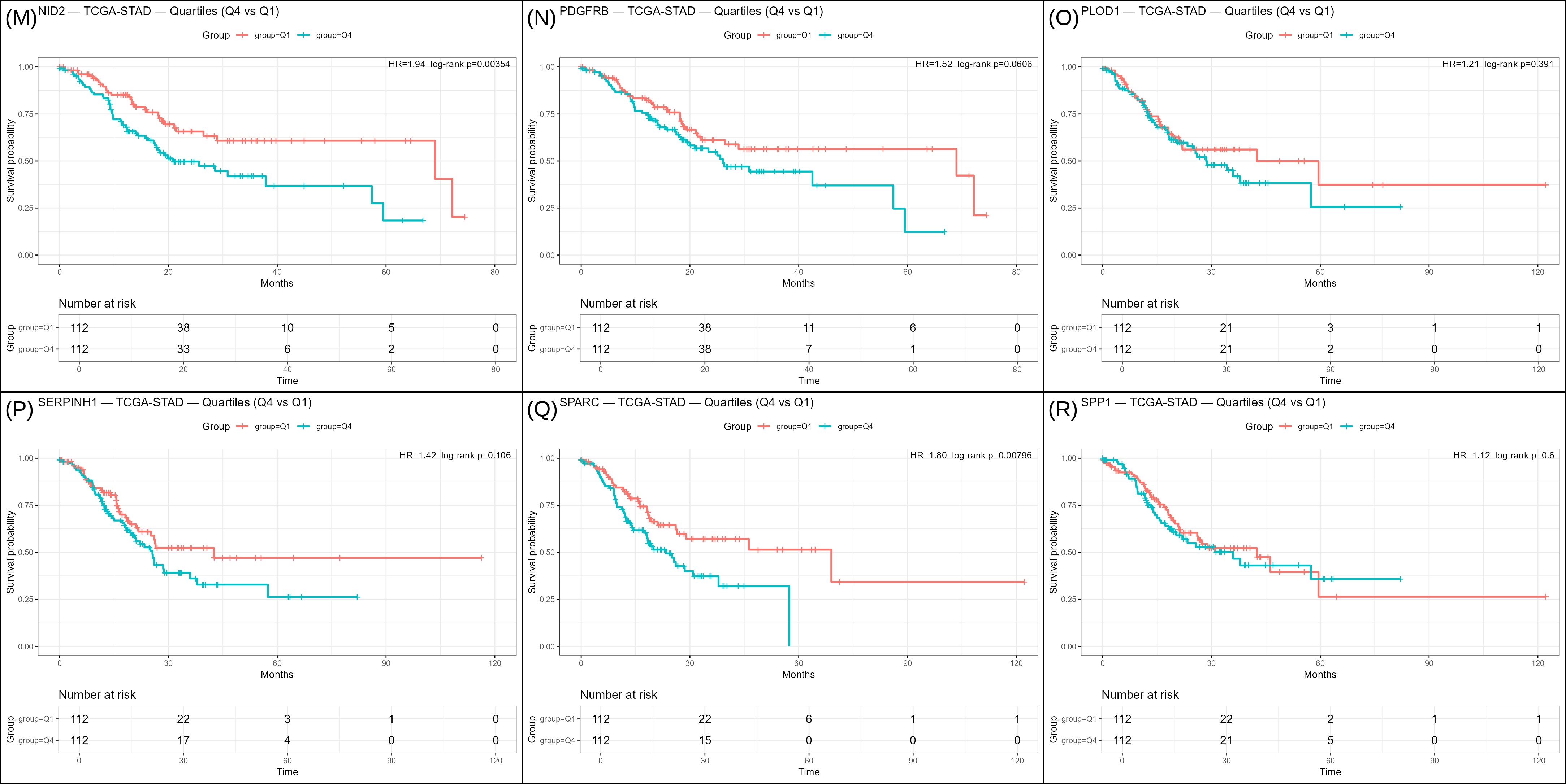


**Supplementary figure 3M-R**- KM plots using the top 25% vs the bottom 25% of expression (Q4 vs Q1) within the TCGA cohort for NID2, PDGFRB, PLOD1, SERPINH1, SPARC, and SPP1 with log-rank p-values, and n-at-risk tables


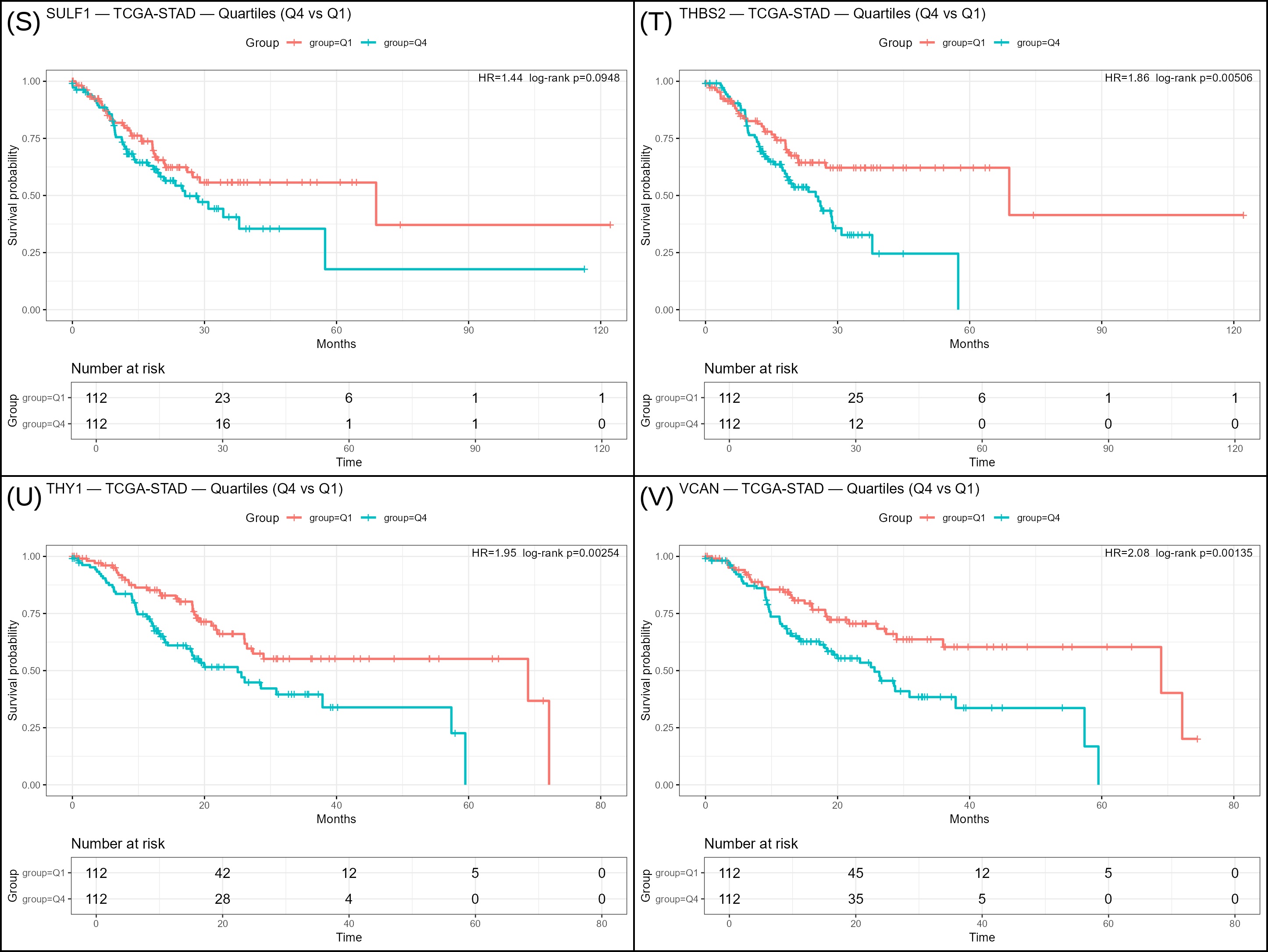


**Supplementary figure 3S-V**- KM plots using the top 25% vs the bottom 25% of expression (Q4 vs Q1) within the TCGA cohort for SULF1, THBS2, THY1, and VCAN with log-rank p-values, and n-at-risk tables

1. **Supplementary figure 4**


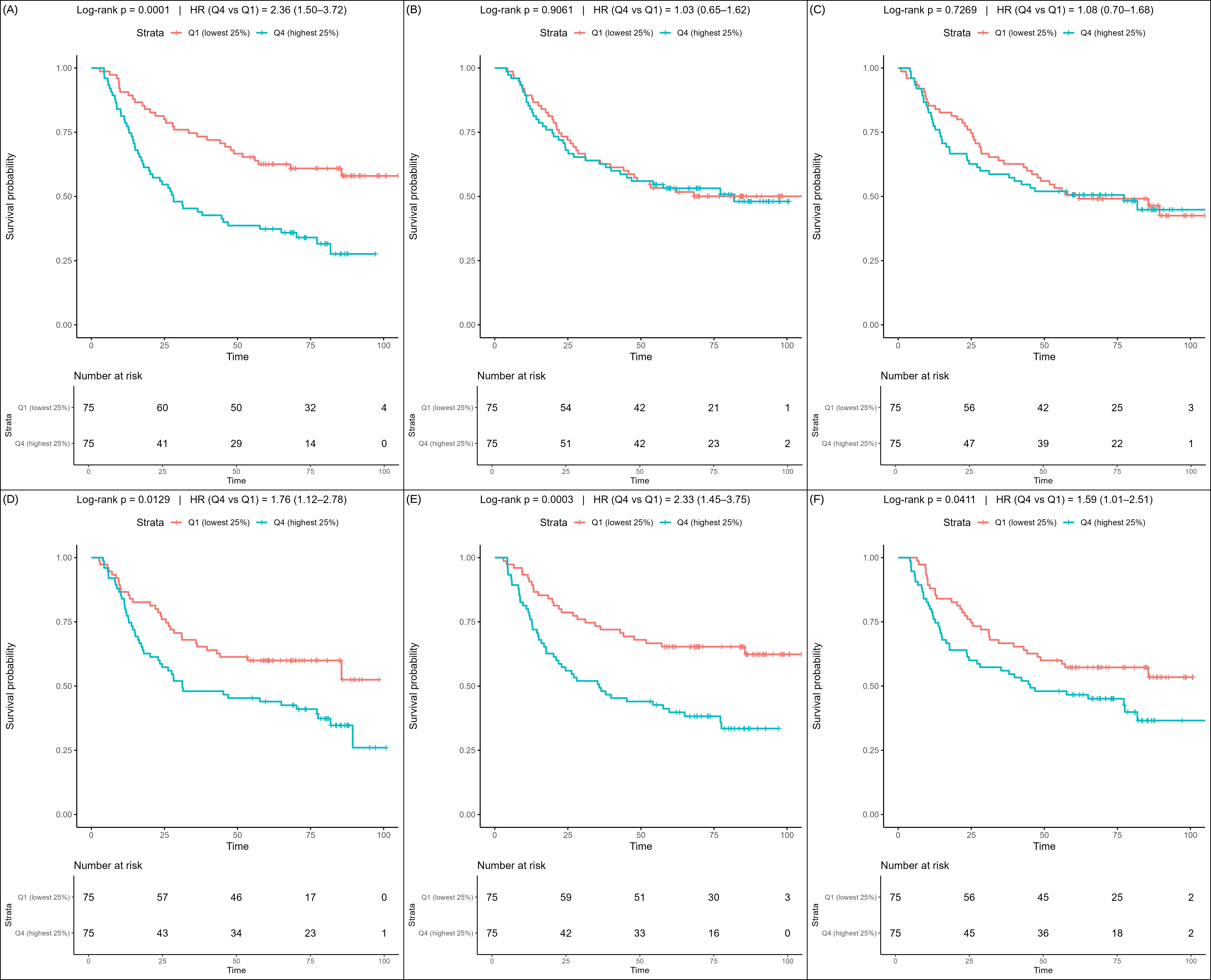


**Supplementary figure 4A-F-** KM plots using the top 25% vs the bottom 25% of expression (Q4 vs Q1) within the GSE62254 cohort for BGN, BMP1, COL12A1, COL18A1, COL1A1, and COL1A2 with log-rank p-values, and n-at-risk tables


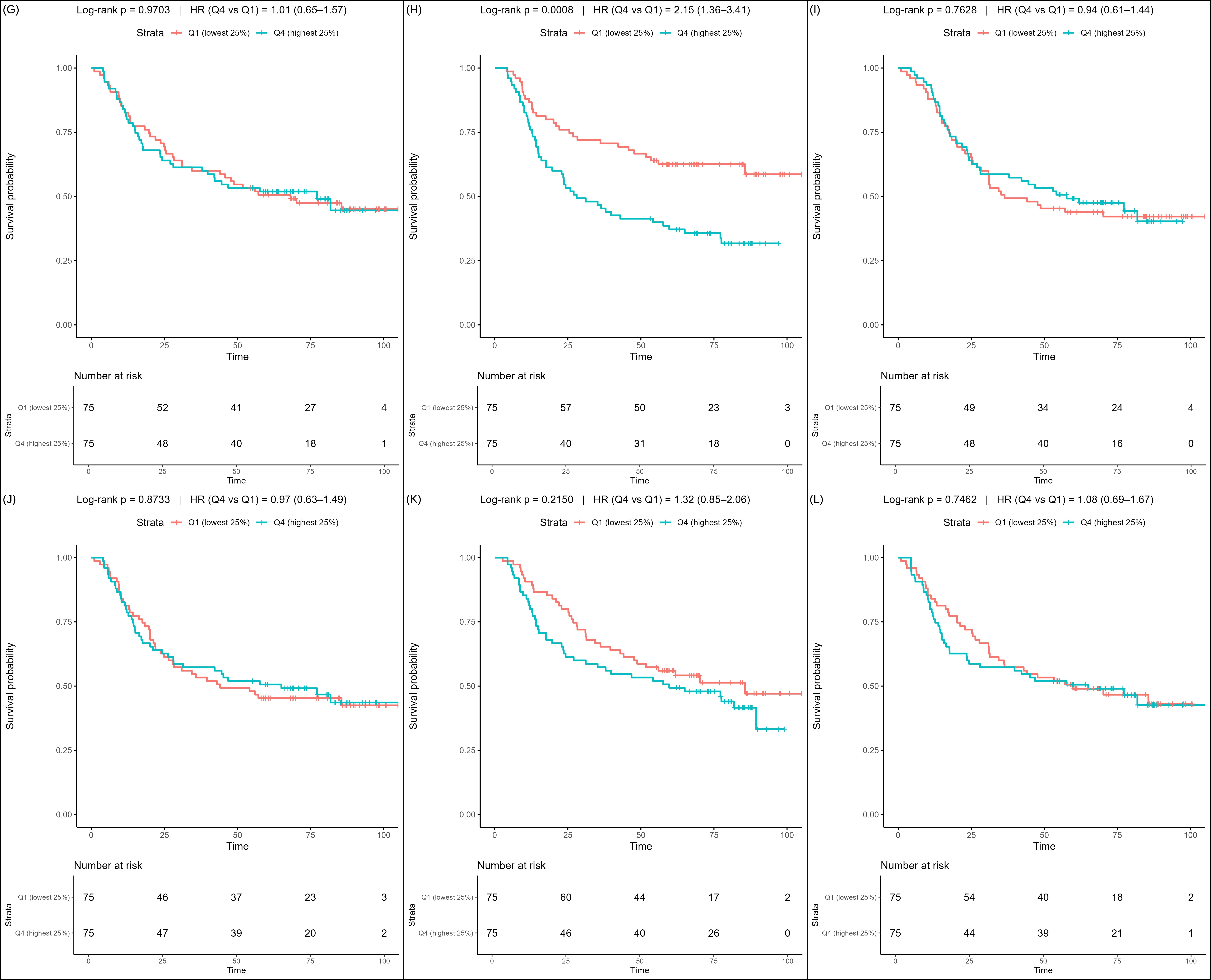


**Supplementary figure 4G-L**- KM plots using the top 25% vs the bottom 25% of expression (Q4 vs Q1) within the GSE62254 cohort for COL4A1, COL5A2, CTSB, FAP, LOXL2, and MMP14 with log-rank p-values, and n-at-risk tables


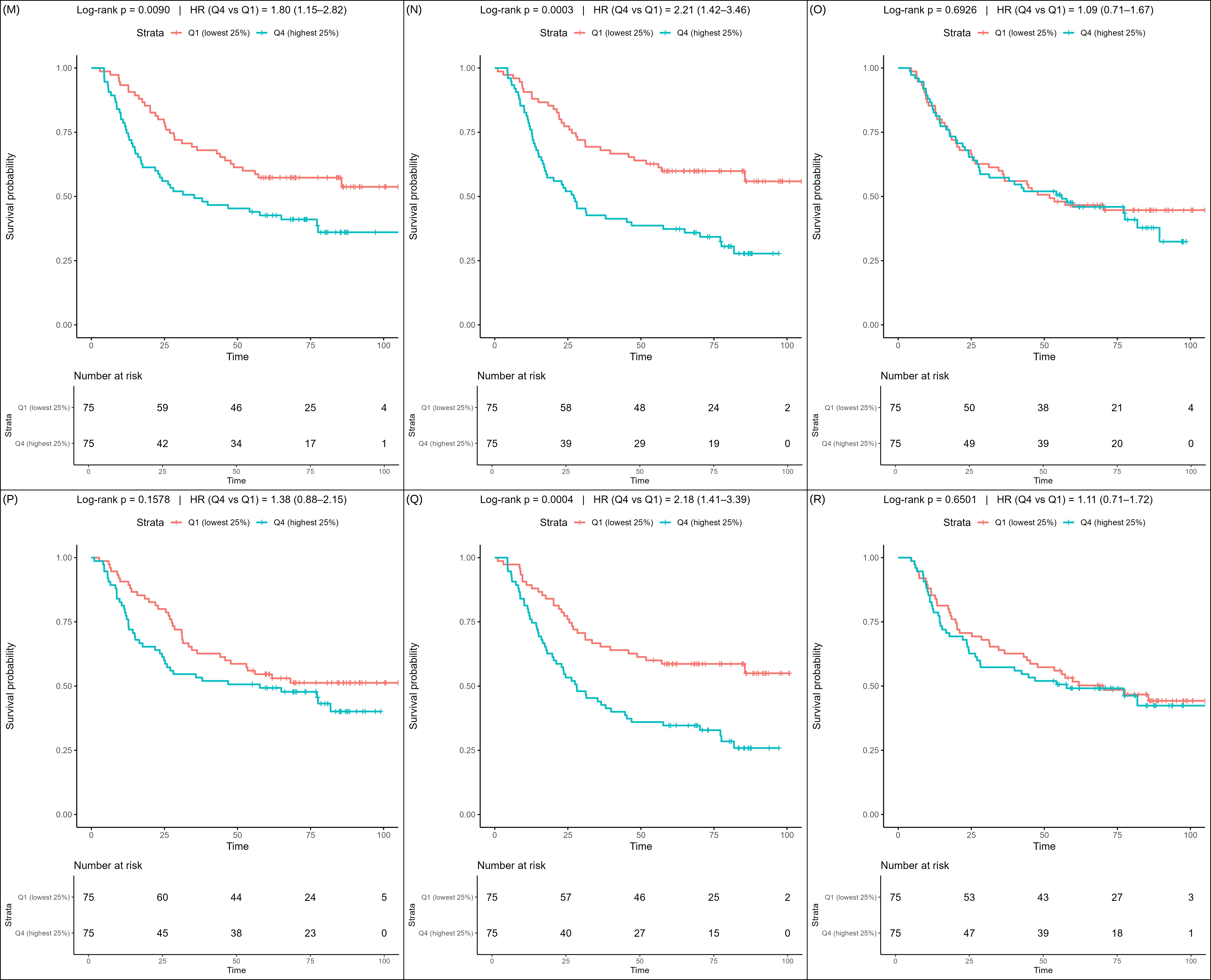


**Supplementary figure 4M-R**- KM plots using the top 25% vs the bottom 25% of expression (Q4 vs Q1) within the GSE62254 cohort for NID2, PDGFRB, PLOD1, SERPINH1, SPARC, and SPP1 with log-rank p-values, and n-at-risk tables


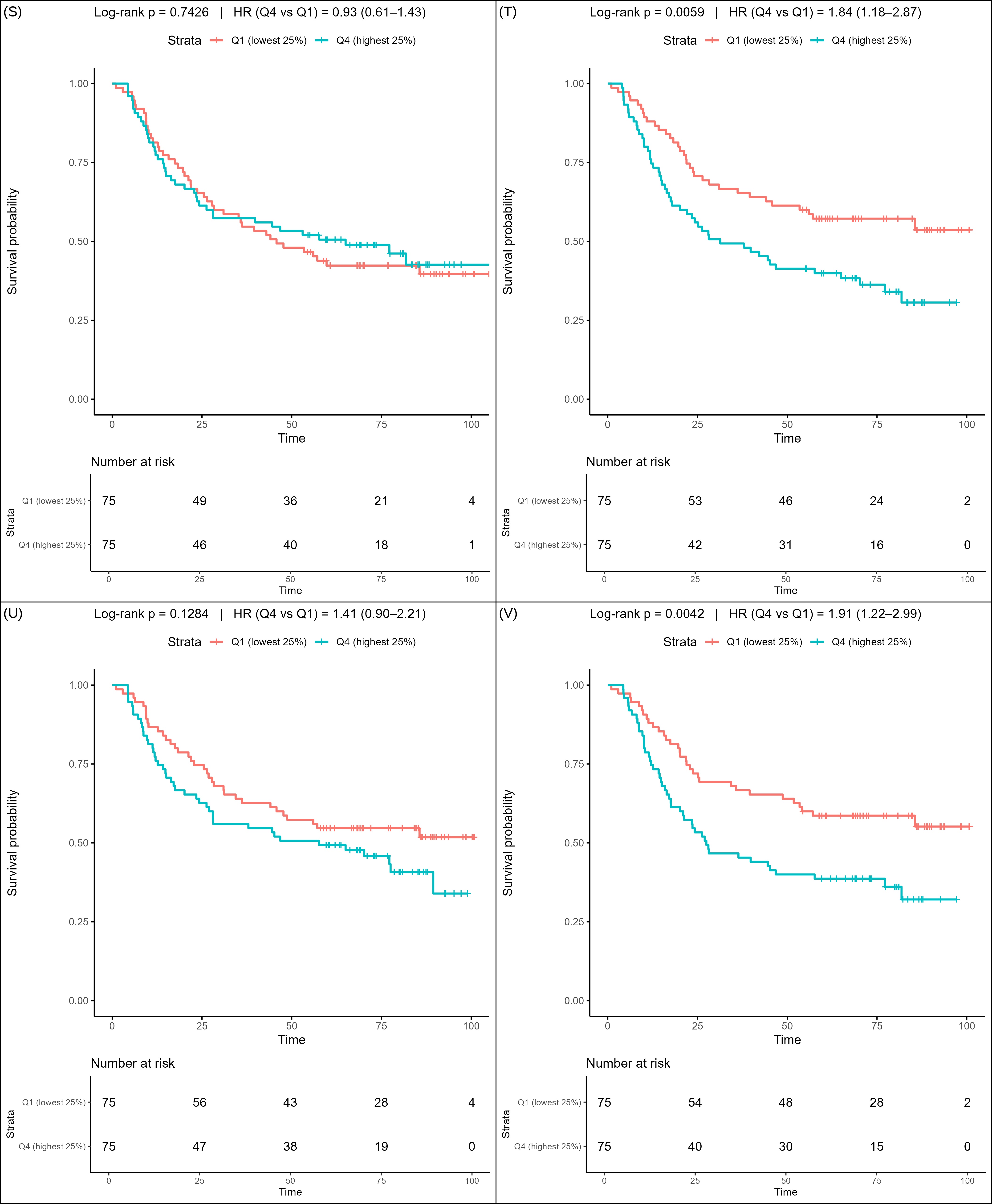


**Supplementary figure 4S-V**- KM plots using the top 25% vs the bottom 25% of expression (Q4 vs Q1) within the GSE62254 cohort for SULF1, THBS2, THY1, and VCAN with log-rank p-values, and n-at-risk tables

1. **Supplementary figure 5**


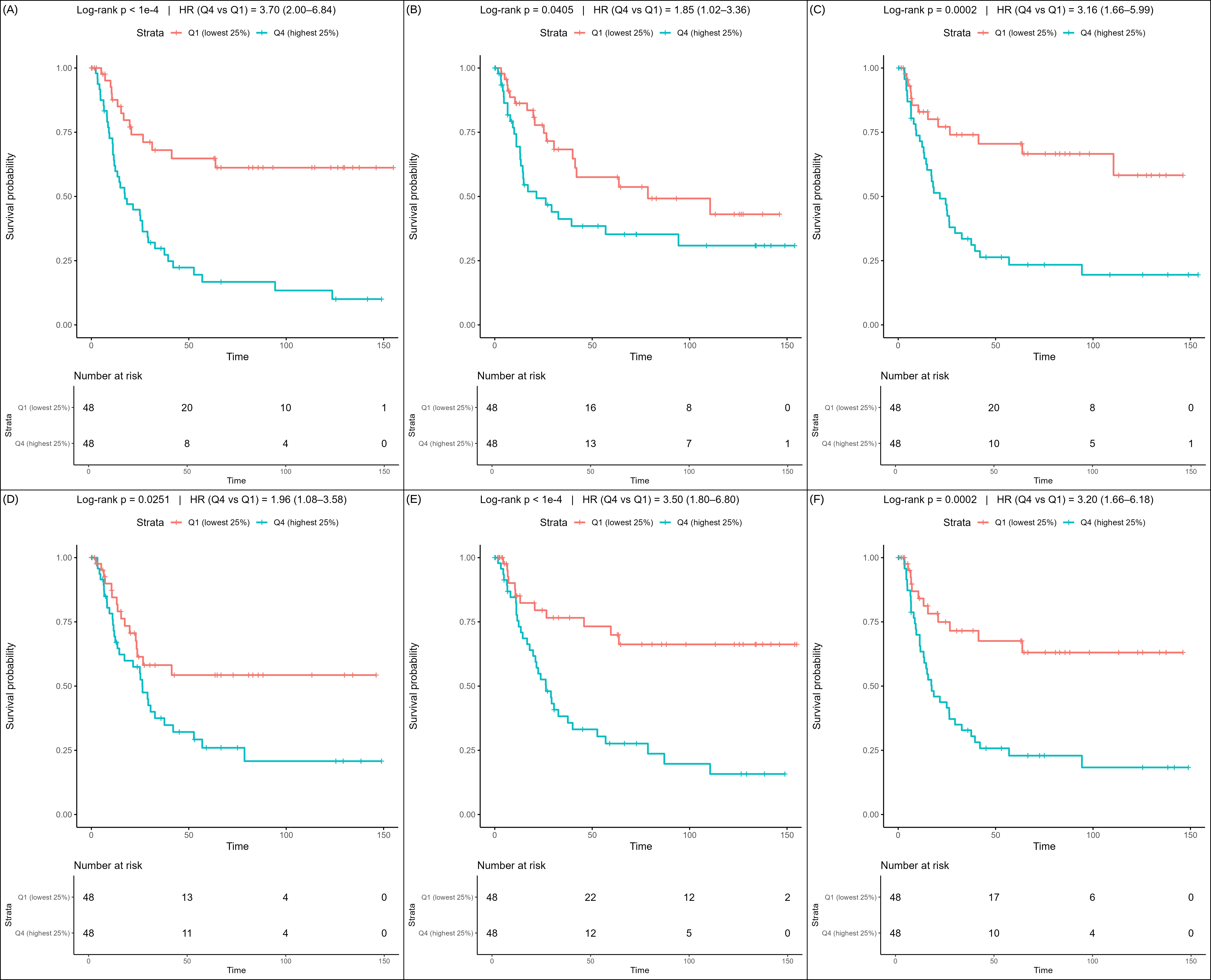


**Supplementary figure 5A-F-** KM plots using the top 25% vs the bottom 25% of expression (Q4 vs Q1) within the GSE15459 cohort for BGN, BMP1, COL12A1, COL18A1, COL1A1, and COL1A2 with log-rank p-values, and n-at-risk tables


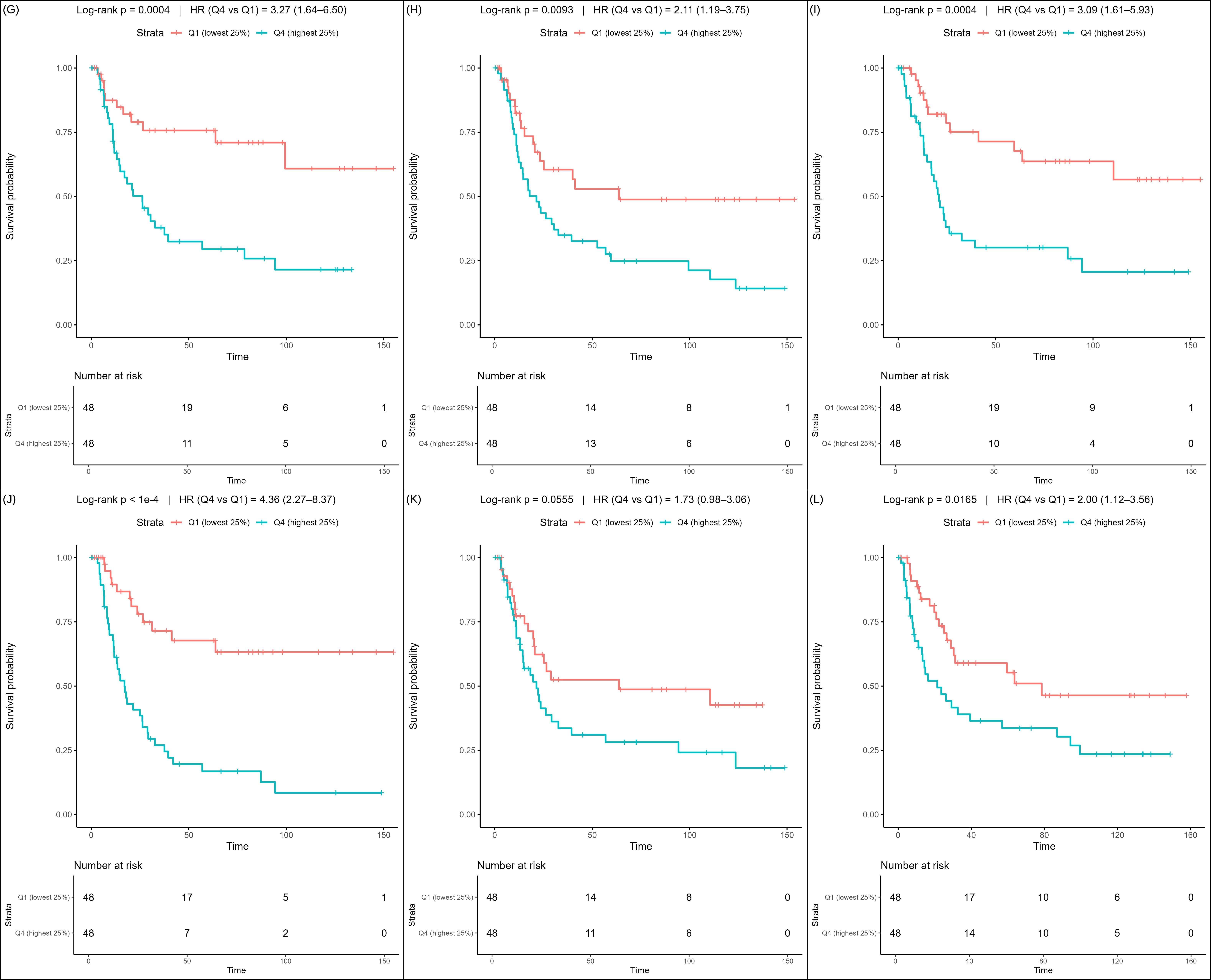


**Supplementary figure 5G-L**- KM plots using the top 25% vs the bottom 25% of expression (Q4 vs Q1) within the GSE15459 cohort for COL4A1, COL5A2, CTSB, FAP, LOXL2, and MMP14 with log-rank p-values, and n-at-risk tables


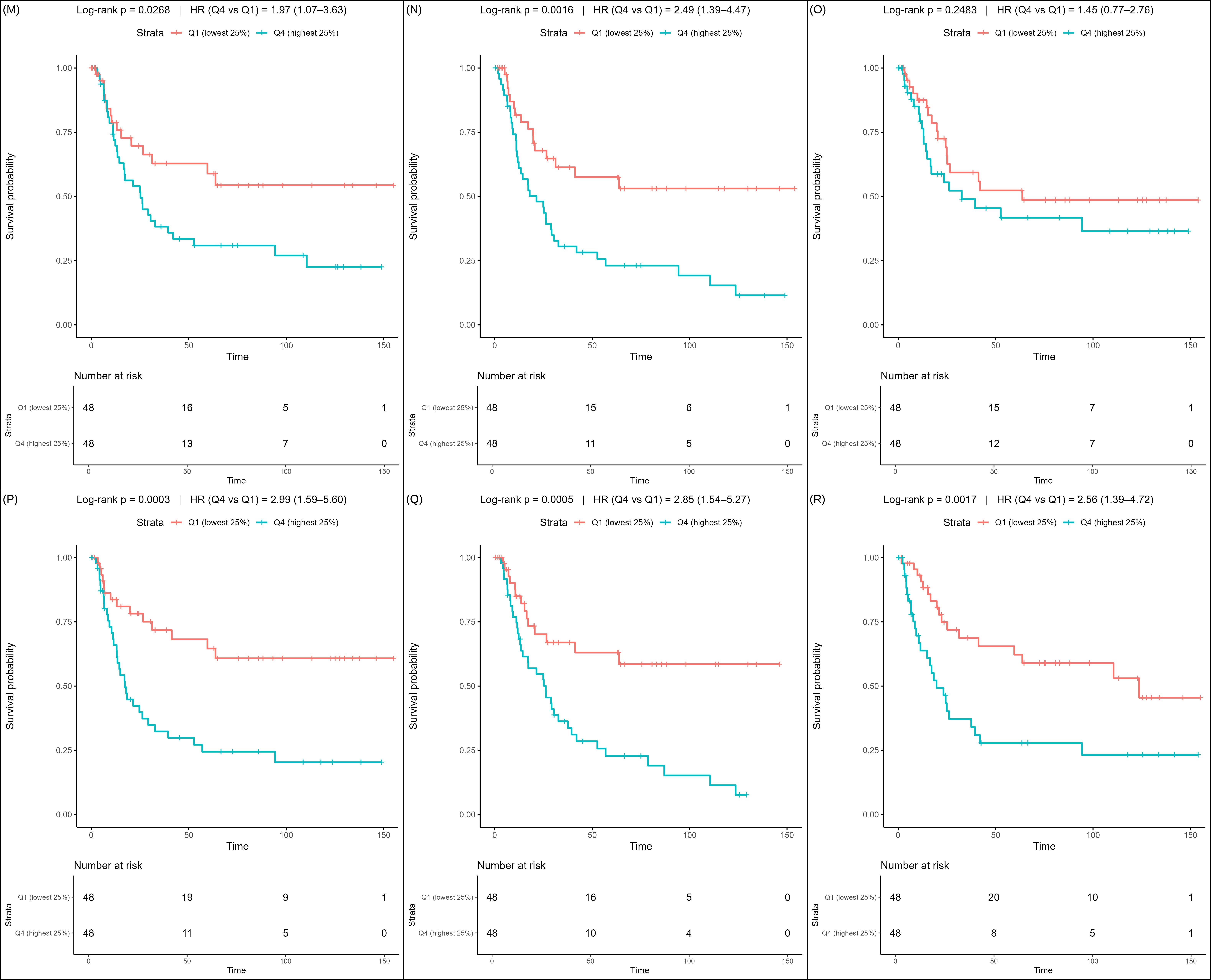


**Supplementary figure 5M-R**- KM plots using the top 25% vs the bottom 25% of expression (Q4 vs Q1) within the GSE15459 cohort for NID2, PDGFRB, PLOD1, SERPINH1, SPARC, and SPP1 with log-rank p-values, and n-at-risk tables


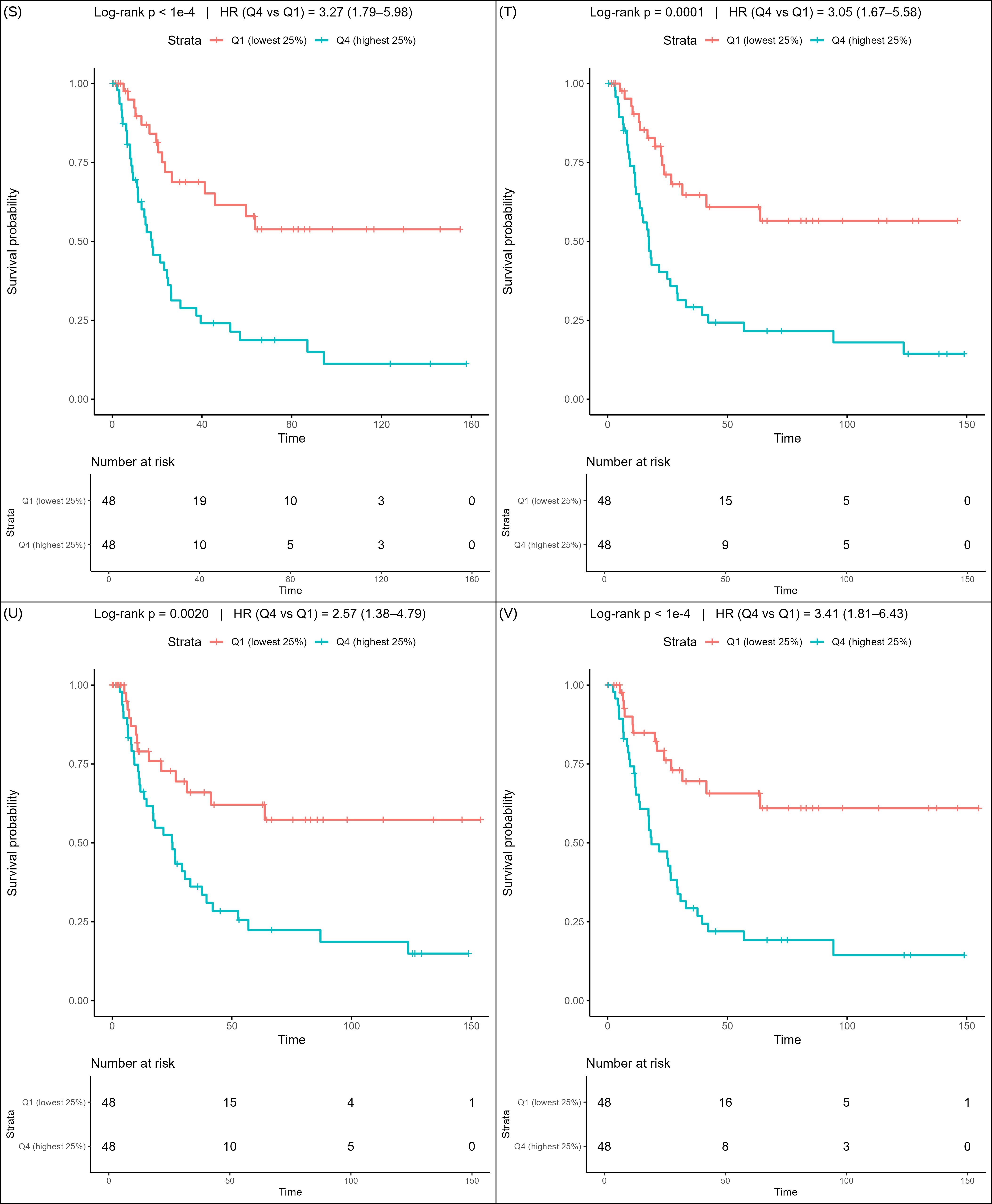


**Supplementary figure 5S-V**- KM plots using the top 25% vs the bottom 25% of expression (Q4 vs Q1) within the GSE15459 cohort for SULF1, THBS2, THY1, and VCAN with log-rank p-values, and n-at-risk tables

1. **Supplementary figure 6**


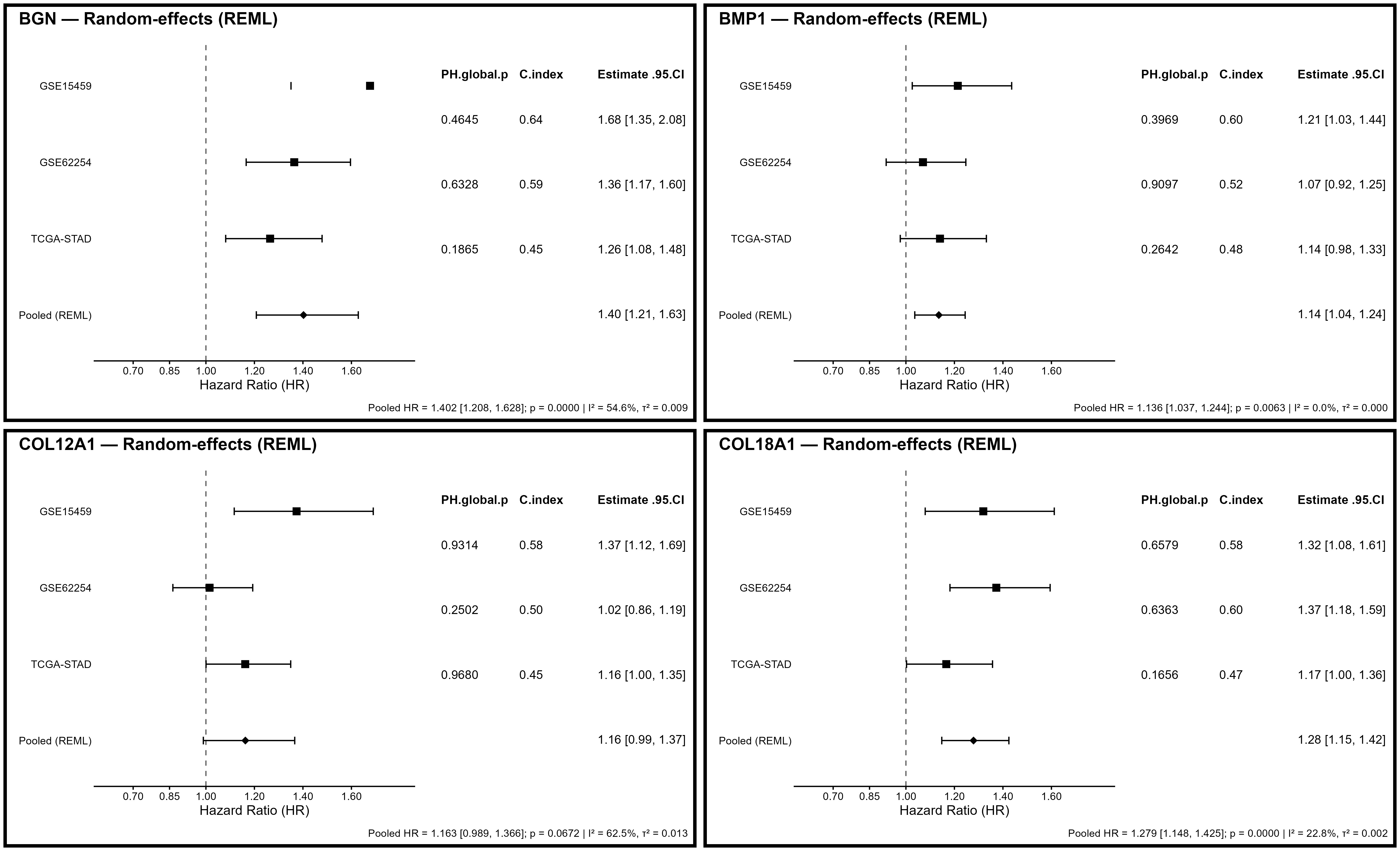

**Supplementary figure 6A**- Forest plots for BGN, BMP1, COL12A1, and COL18A1 with PH.global.p, c-index, and estimate 95% CI from all three cohorts used in survival analysis.


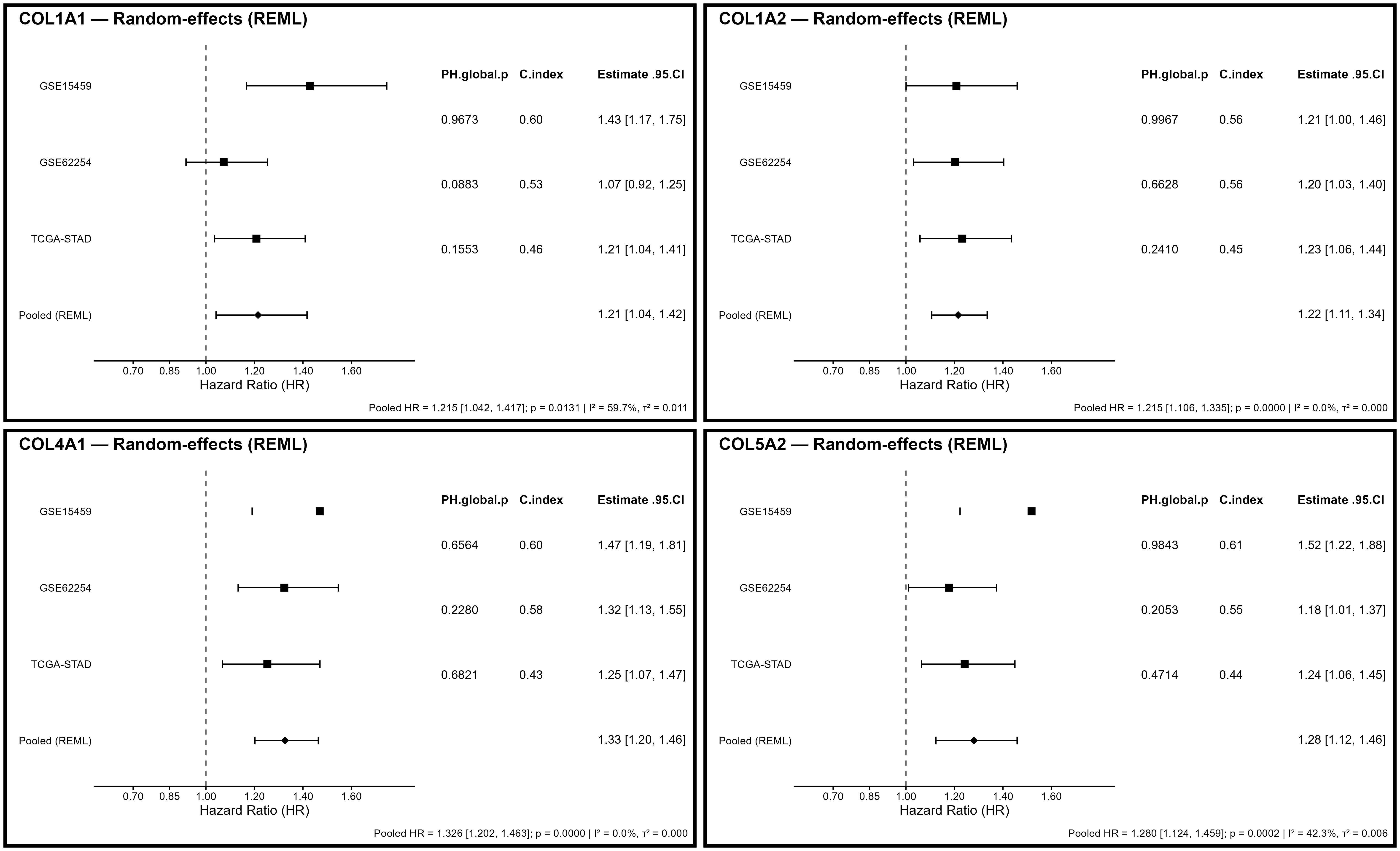


**Supplementary Figure 6B**- Forest plots for COL1A1, COL1A2, COL4A1, and COL5A2 with PH.global.p, c-index, and estimate 95% CI from all three cohorts used in survival analysis.


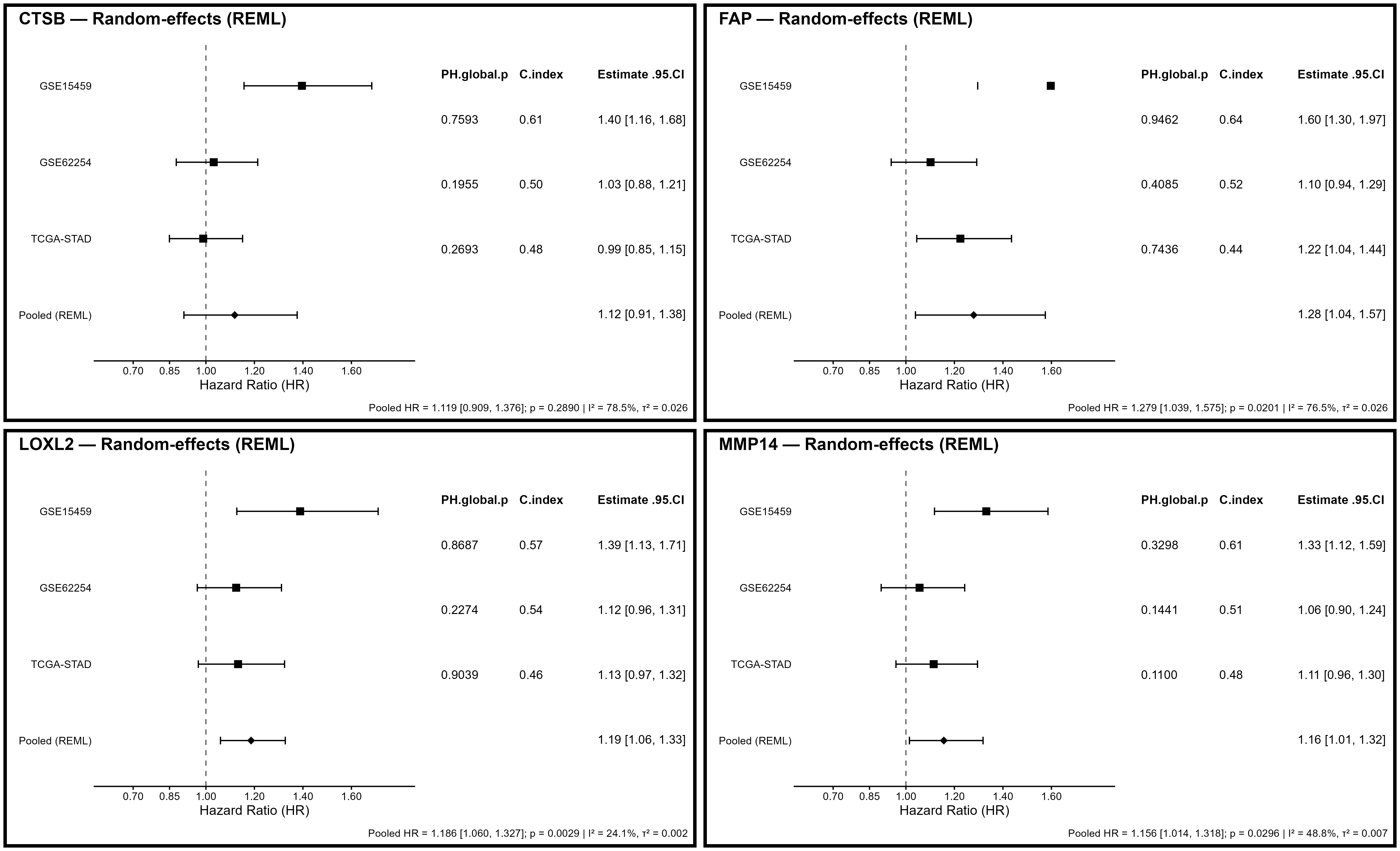


**Supplementary figure 6C**- Forest plots for CTSB, FAP, LOXL2, and MMP14 with PH.global.p, c-index, and estimate 95% CI from all three cohorts used in survival analysis.


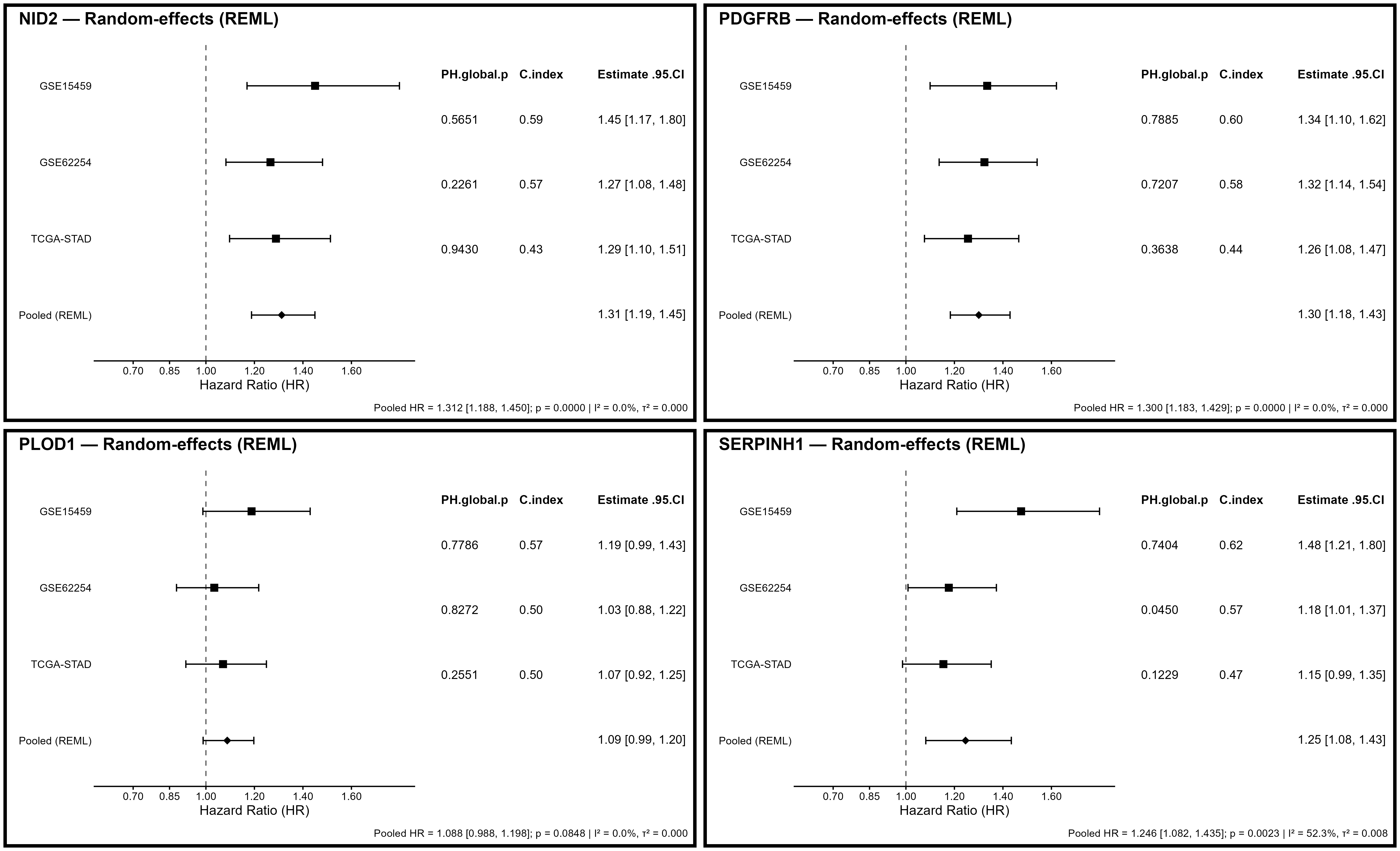


**Supplementary figure 6D**- Forest plots for NID2, PDGFRB, PLOD1, and SERPINH1with PH.global.p, c-index, and estimate 95% CI from all three cohorts used in survival analysis.


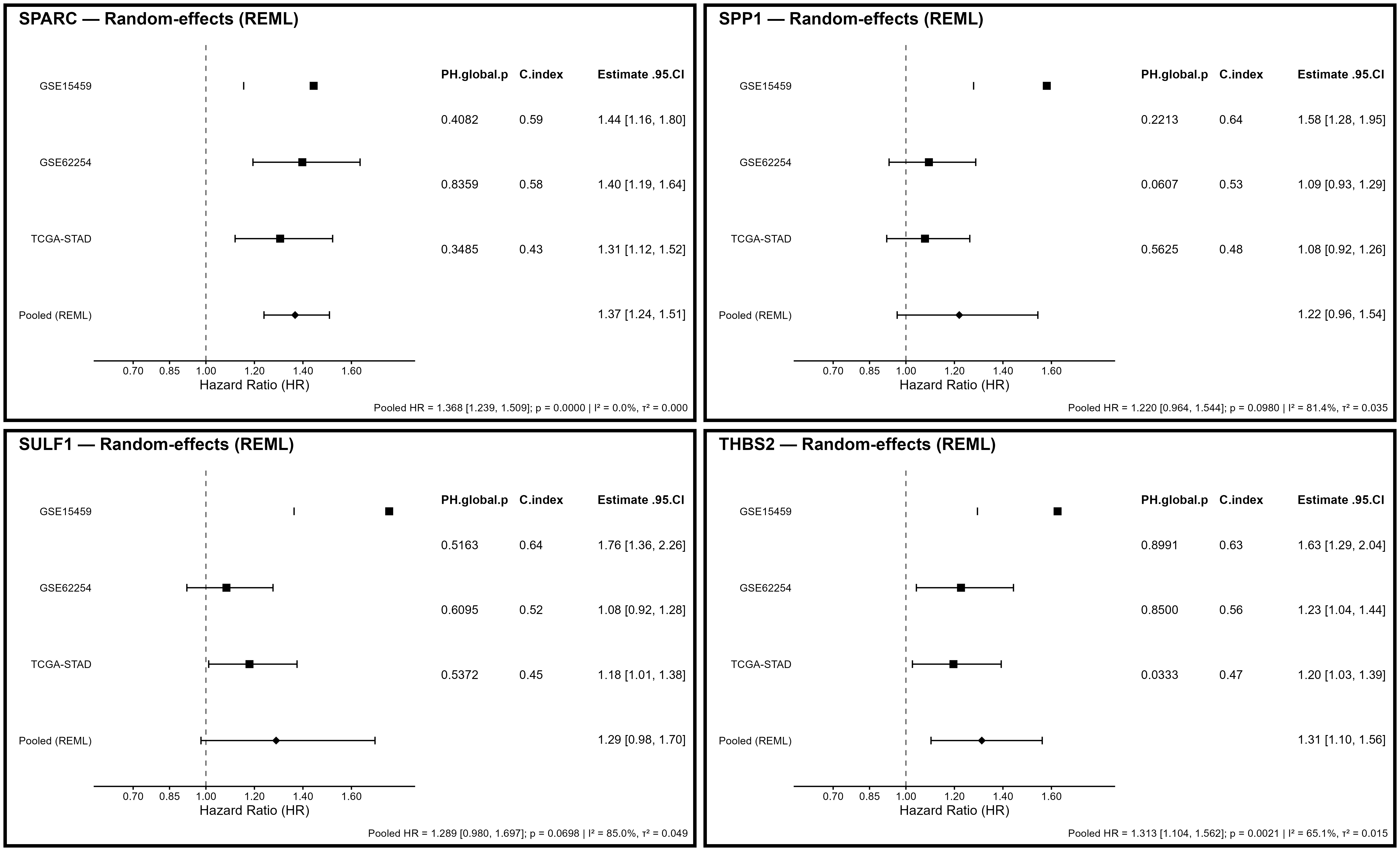


**Supplementary figure 6E-** Forest plots for SPARC, SPP1, SULF1, and THBS2 with PH.global.p, c-index, and estimate 95% CI from all three cohorts used in survival analysis.


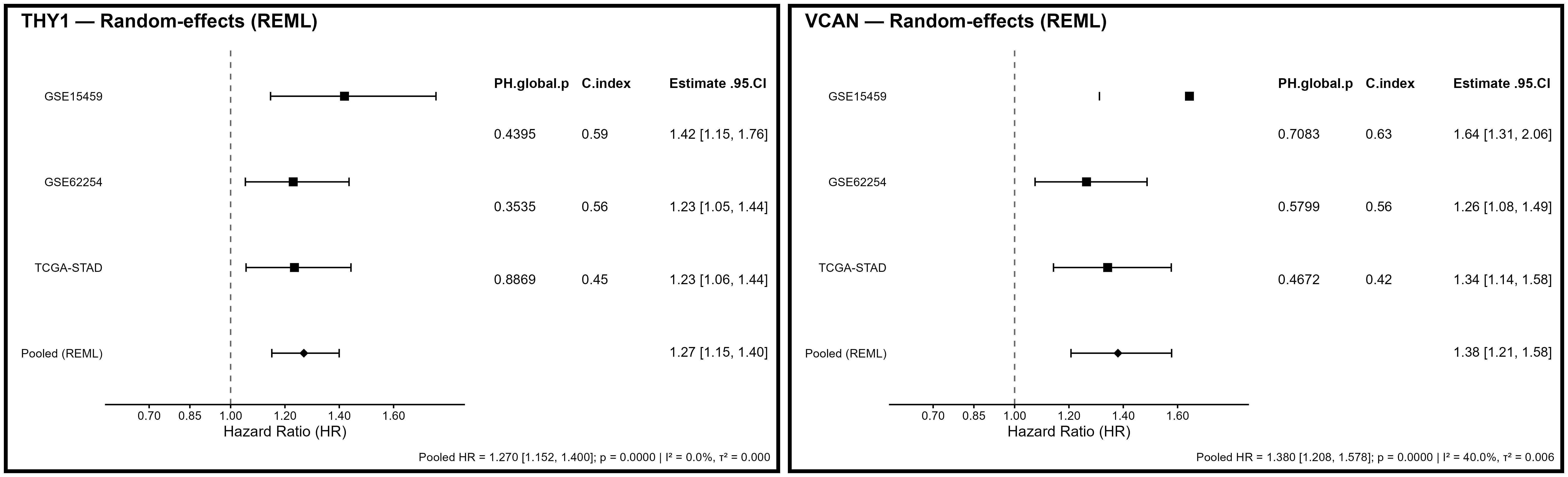


**Supplementary figure 6F**- Forest plots for THY1 and VCAN with PH.global.p, c-index, and estimate 95% CI from all three cohorts used in survival analysis.
