# Supplementary material for: Identification and validation of tumor microenvironment-related therapeutic targets in gastric cancer using integrated multi-omics and molecular docking approaches
Source: Front Bioinform. 2025 Dec 10;5:1654326. doi: 10.3389/fbinf.2025.1654326 (PMC12727970; doi:10.3389/fbinf.2025.1654326)
Supplement: Supplementary file 9 [file DataSheet1.docx]

Supplementary FIGURES_1&2

Identification And Validation of Tumor Microenvironment-Related Therapeutic Targets in Gastric Cancer Using Integrated Multiomics and Molecular Docking Approaches

1. **Supplementary Figure 1**


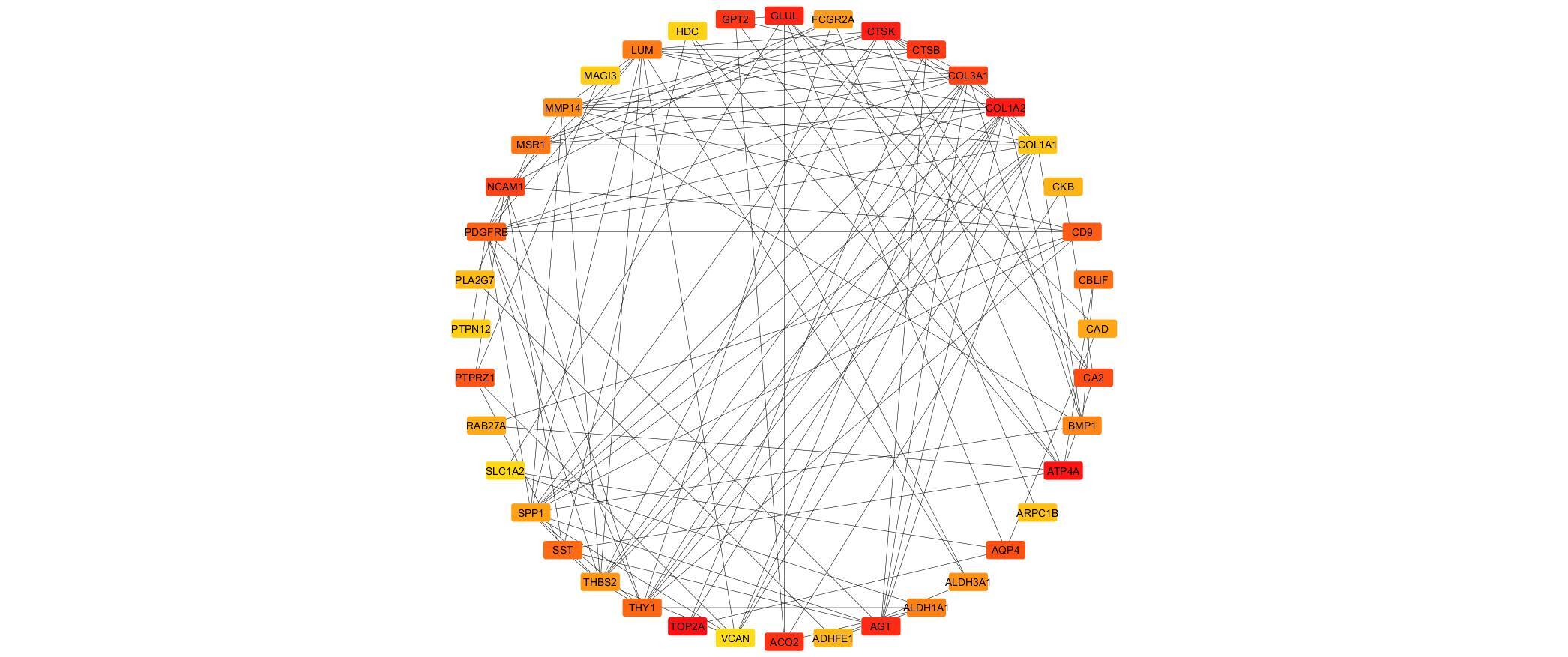


**Supplementary Figure 1A:** Top 40 genes from the Betweenness method in Cytohubba.


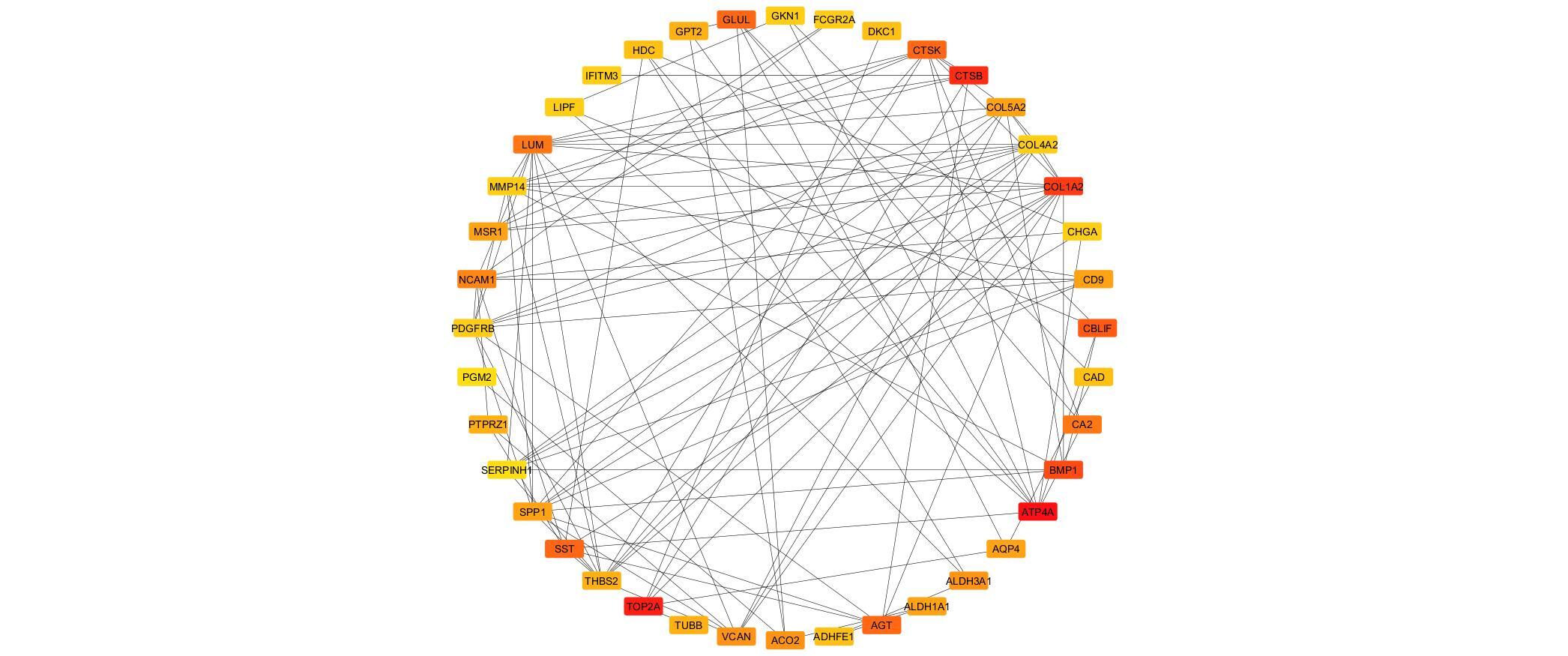


**Supplementary Figure 1B**- Top 40 genes from the Bottleneck method in Cytohubba


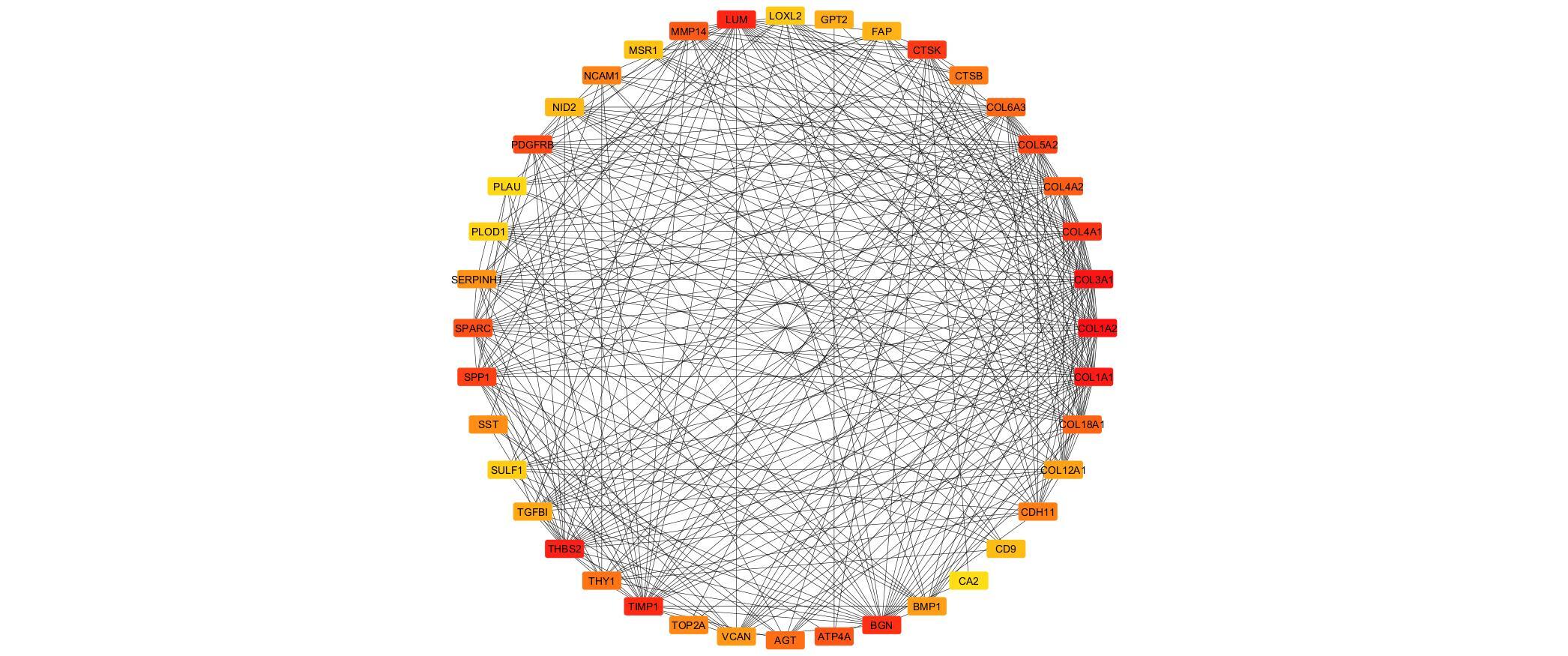


**Supplementary Figure 1C**- Top 40 genes from the Closeness method in Cytohubba


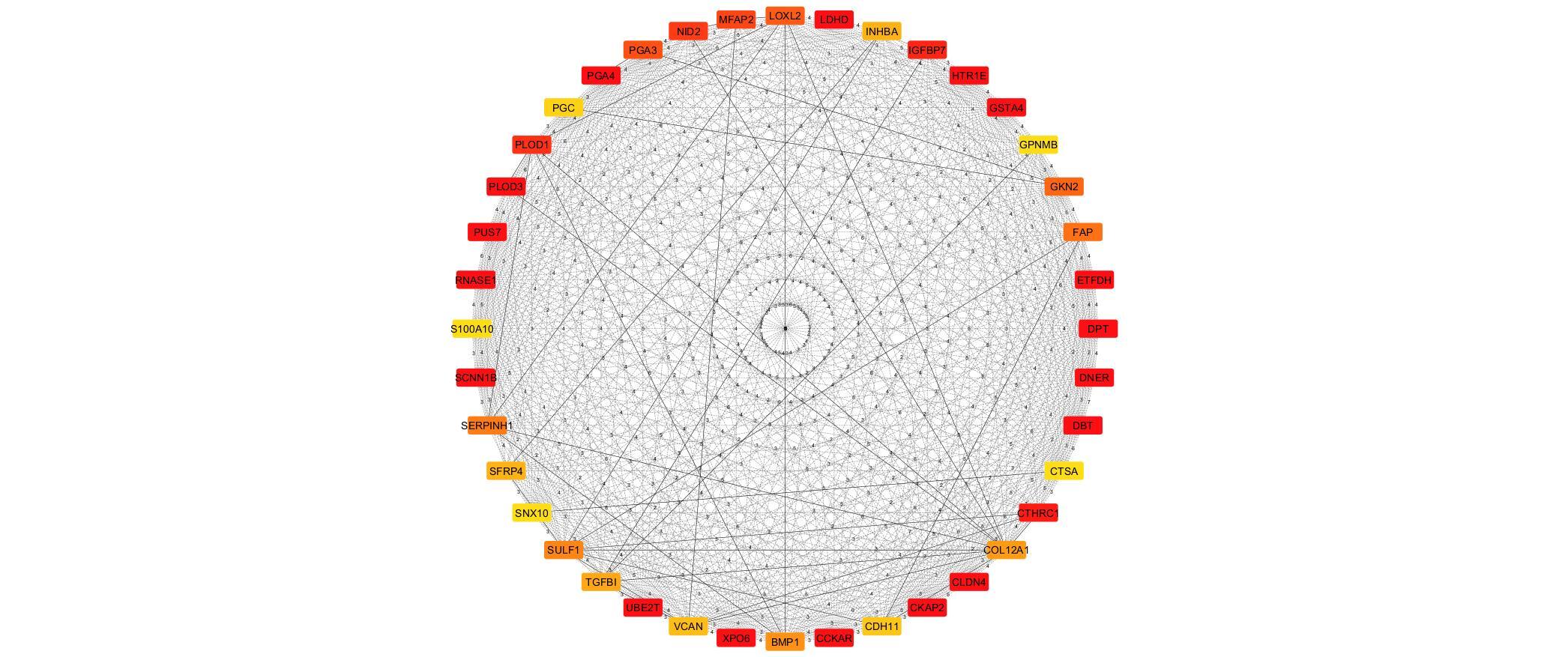


**Supplementary Figure 1D**- Top 40 genes from the Clustering Coefficient method in Cytohubba


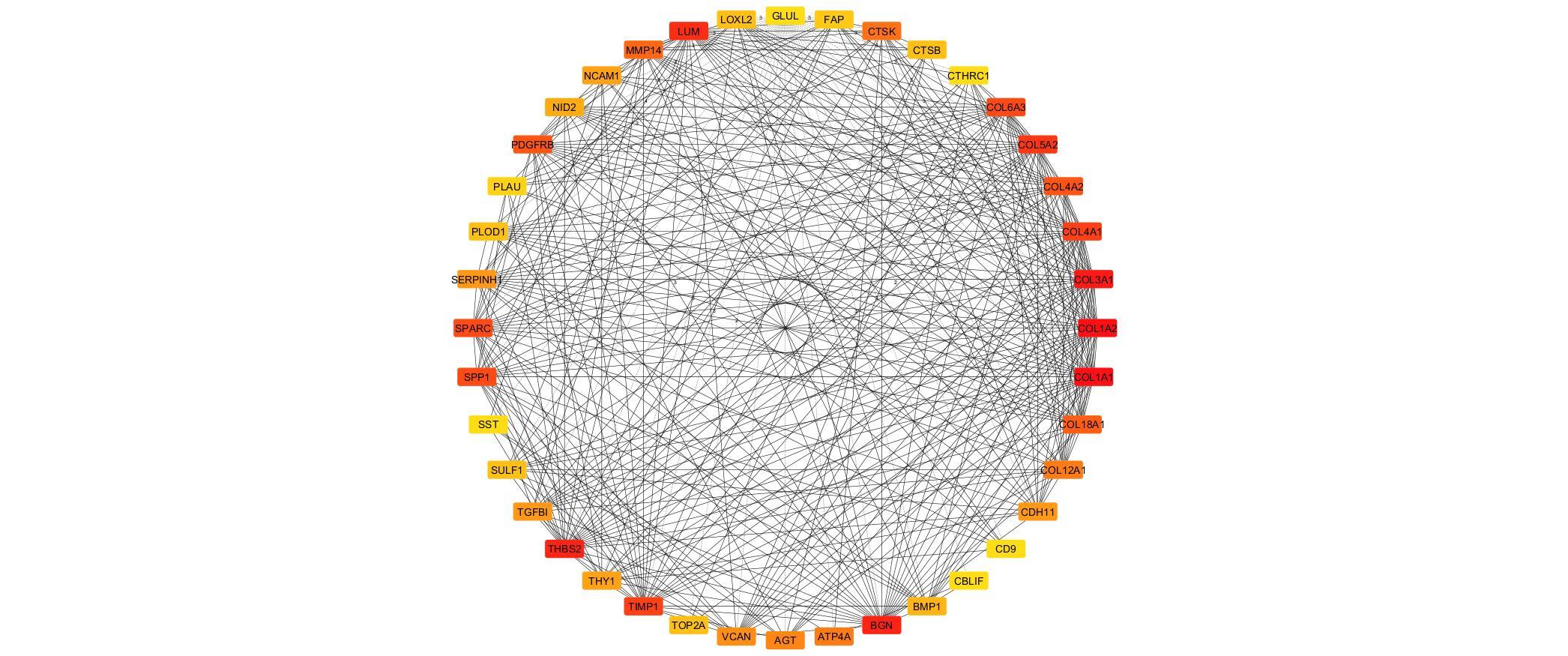


**Supplementary Figure 1E-** Top 40 genes from the Degree method in Cytohubba


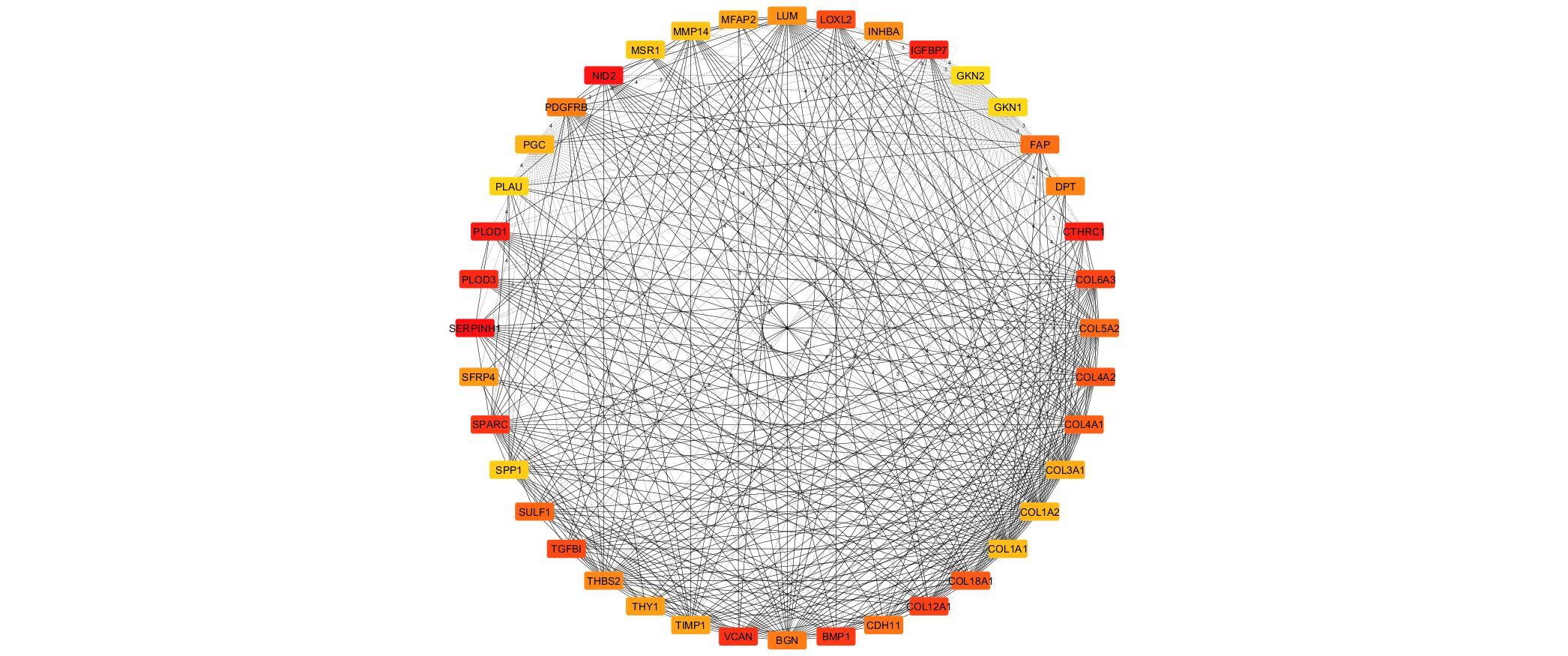


**Supplementary Figure 1F**- Top 40 genes from the DMNC method in Cytohubba

**
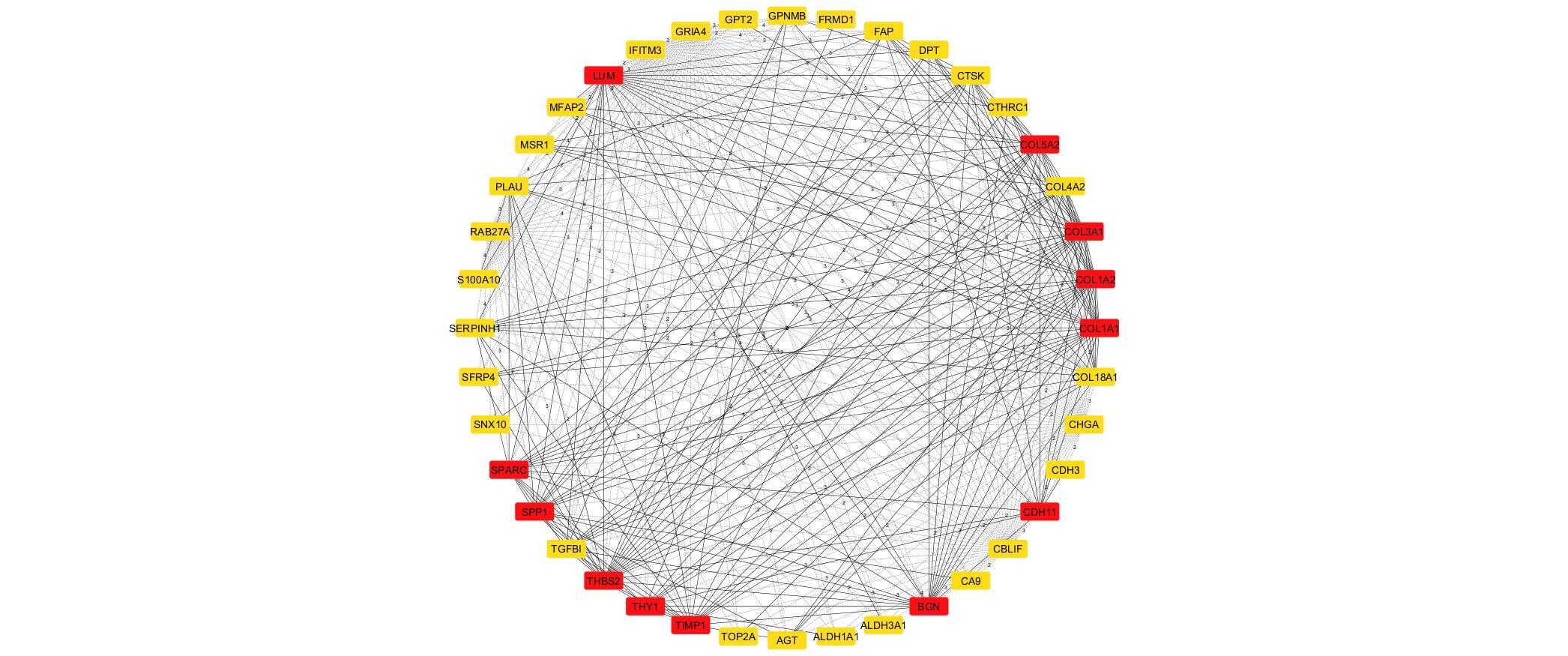
**

**Supplementary Figure 1G**- Top 40 genes from the EcCentricity method in Cytohubba


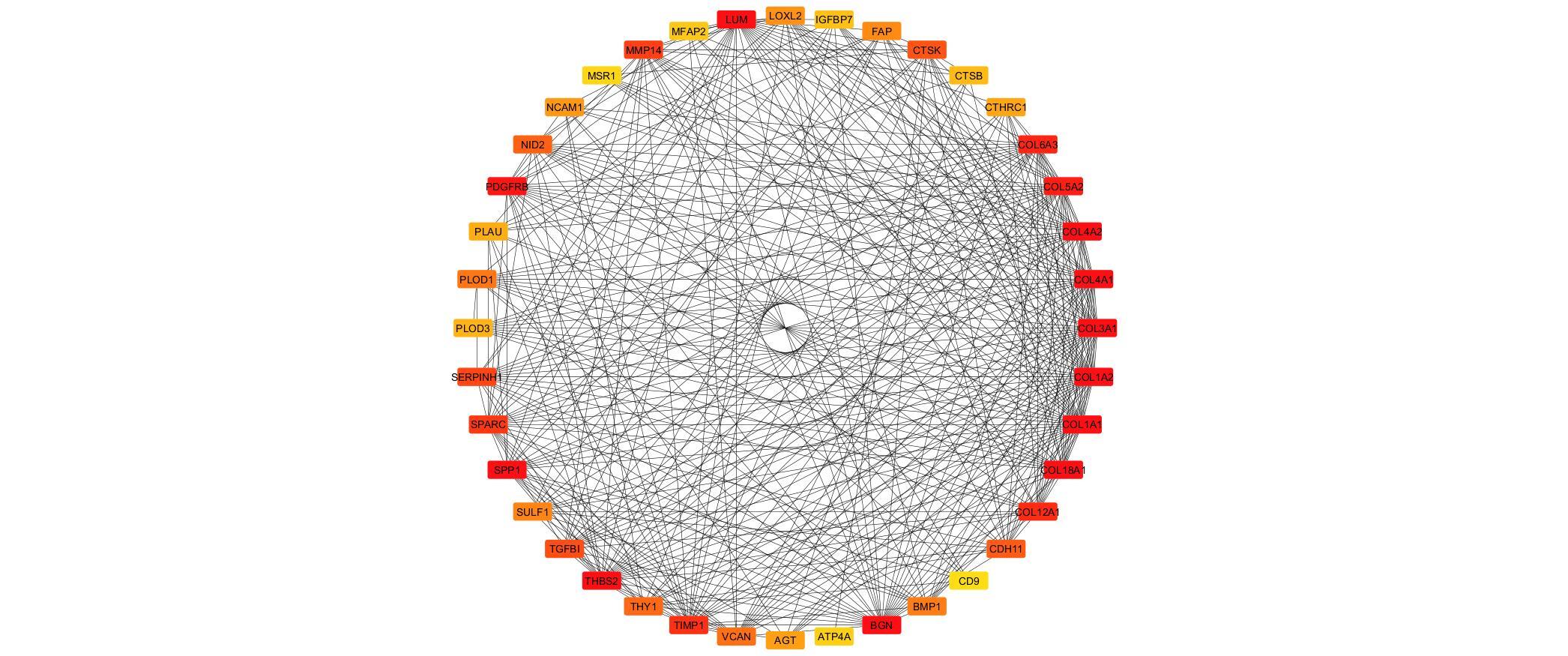


**Supplementary Figure 1H**- Top 40 genes from the EPC method in Cytohubba


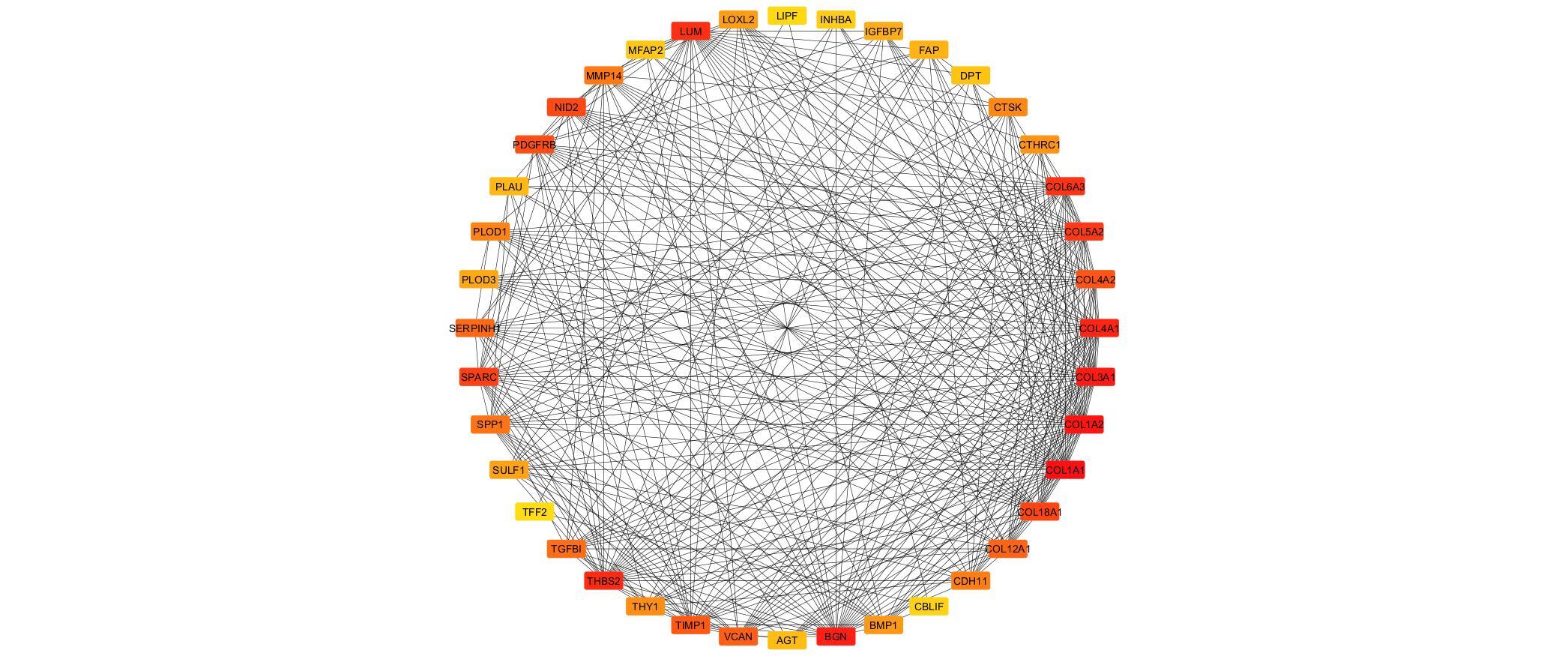


**Supplementary Figure 1I**- Top 40 genes from the MCC method in Cytohubba

**
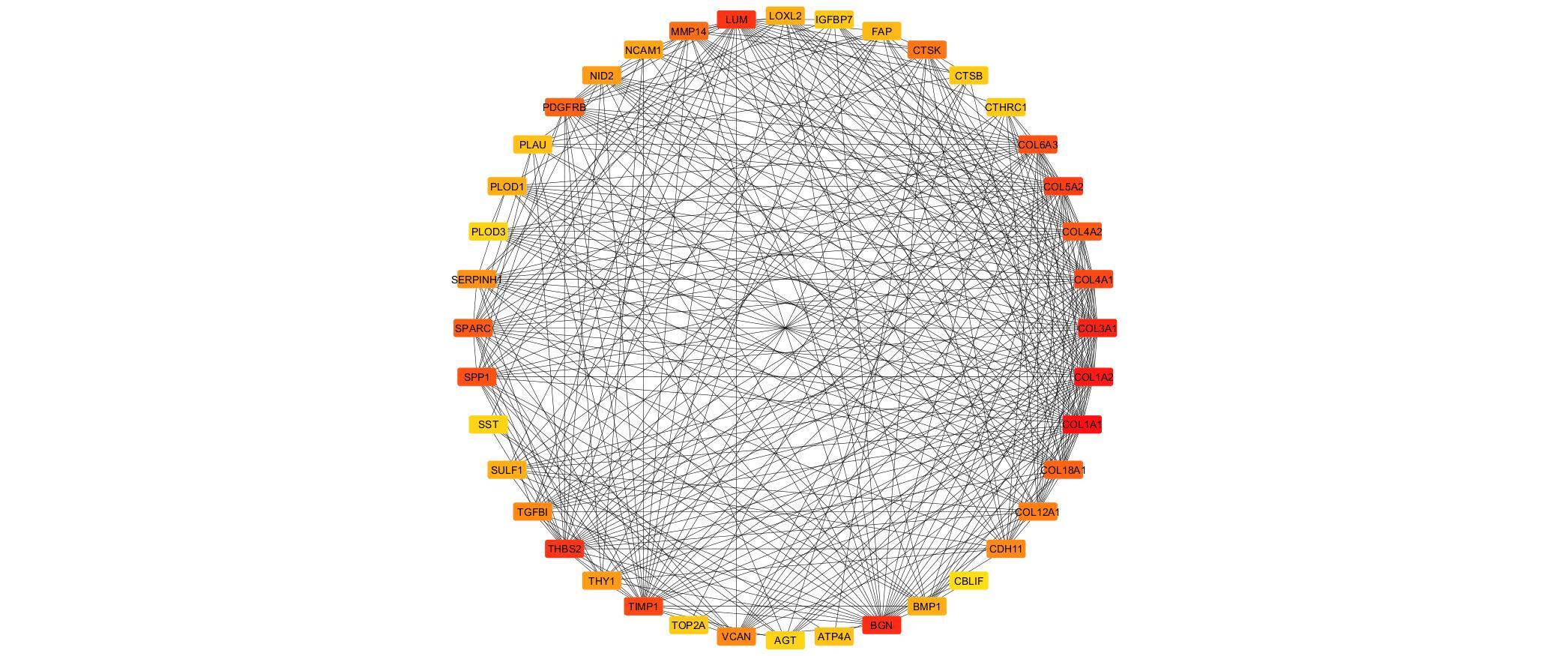
**

**Supplementary Figure 1J**- Top 40 genes from the MNC method in Cytohubba


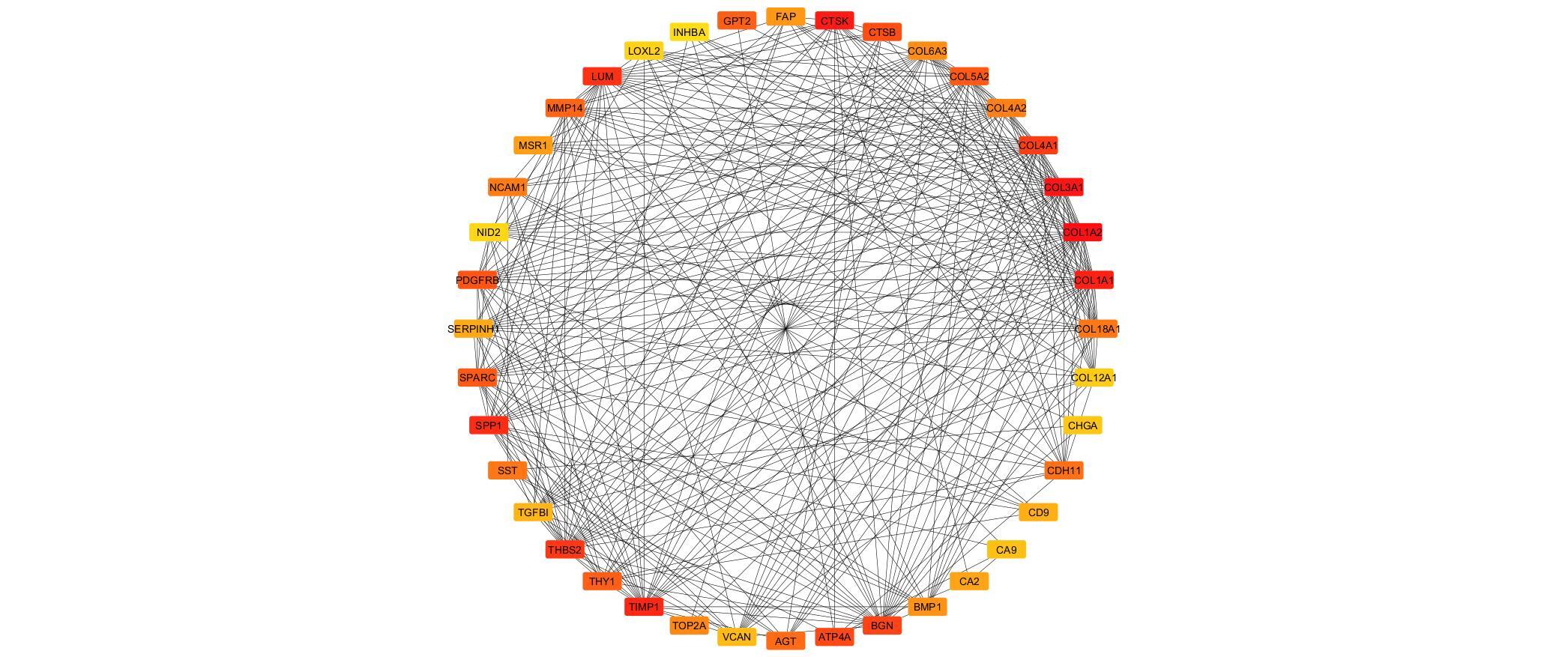


**Supplementary Figure 1K**- Top 40 genes from the Radiality method in Cytohubba


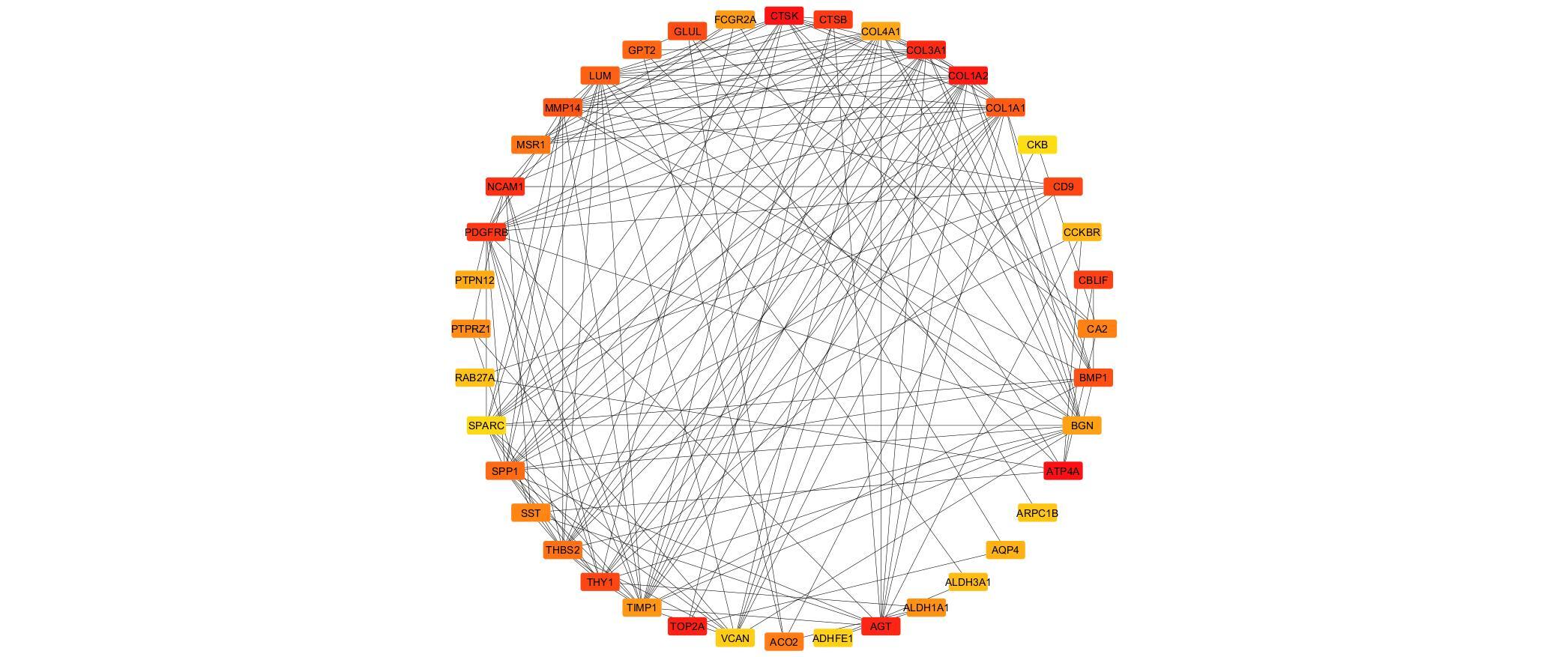


**Supplementary Figure 1L**- Top 40 genes from the Stress method in Cytohubba

1. **Supplementary figure 2**


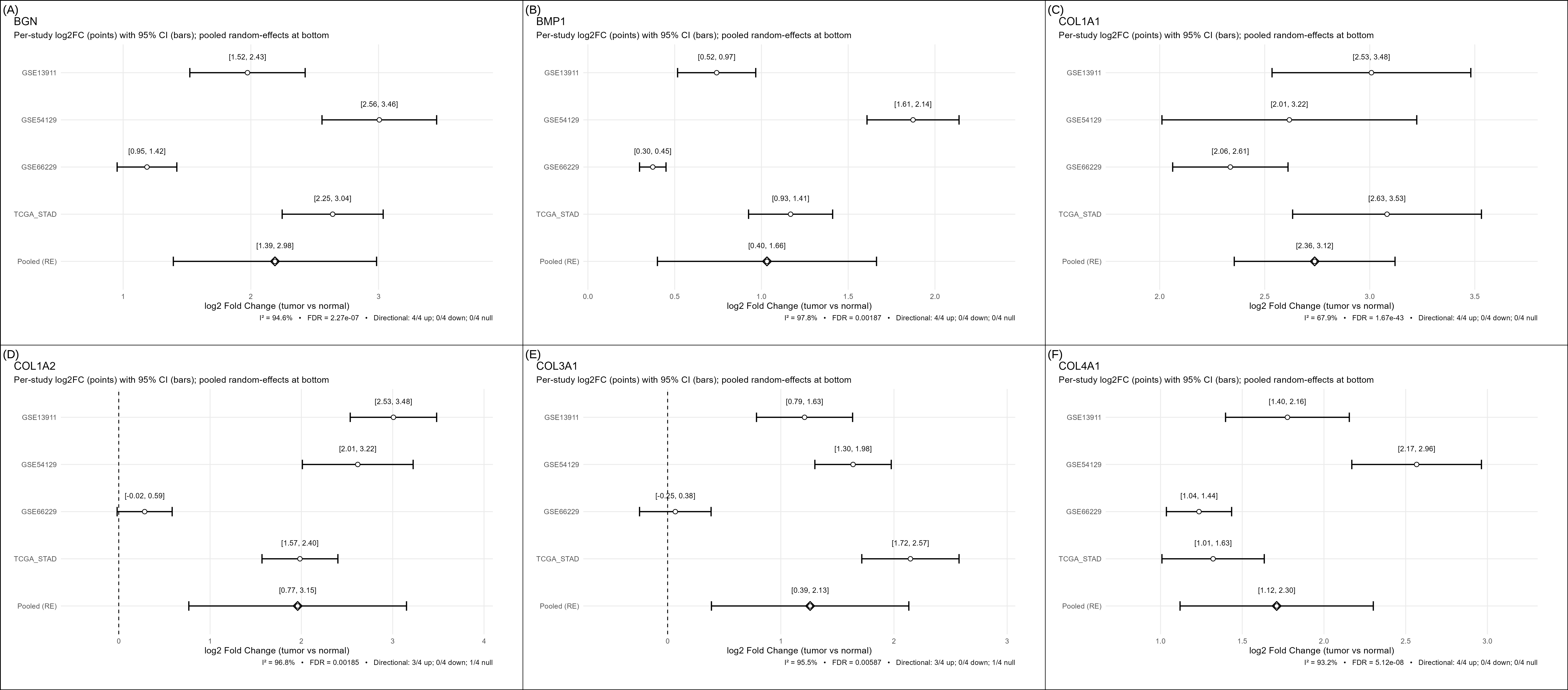


**Supplementary figure 2A-F**: Forest plots of BGN, BMP1, COL1A1, COL1A2, COL3A1, and COL4A1 with I^2^, FDR and directional information


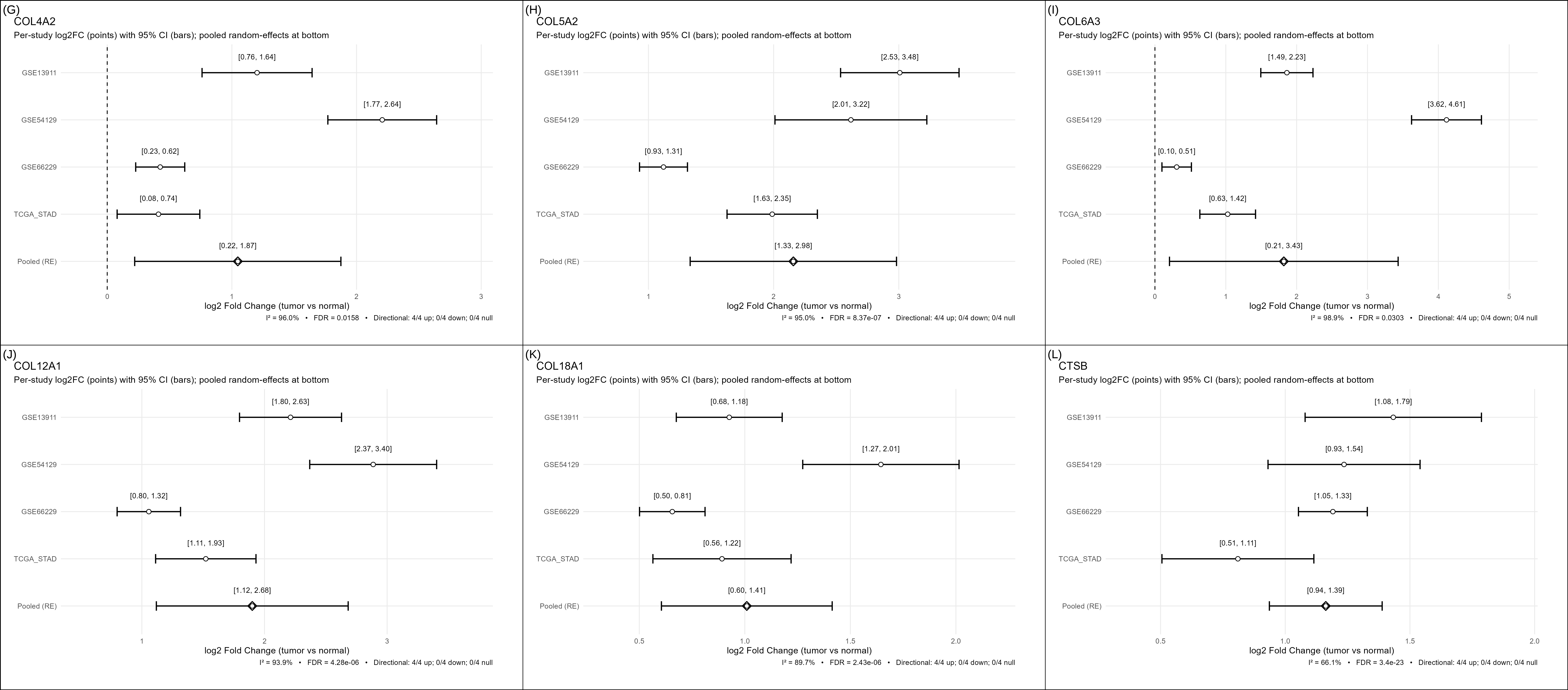


**Supplementary figure 2G-L**: Forest plots of COL4A2, COL5A2, COL6A3, COL12A1, COL18A1, and CTSB with I^2^, FDR and directional information


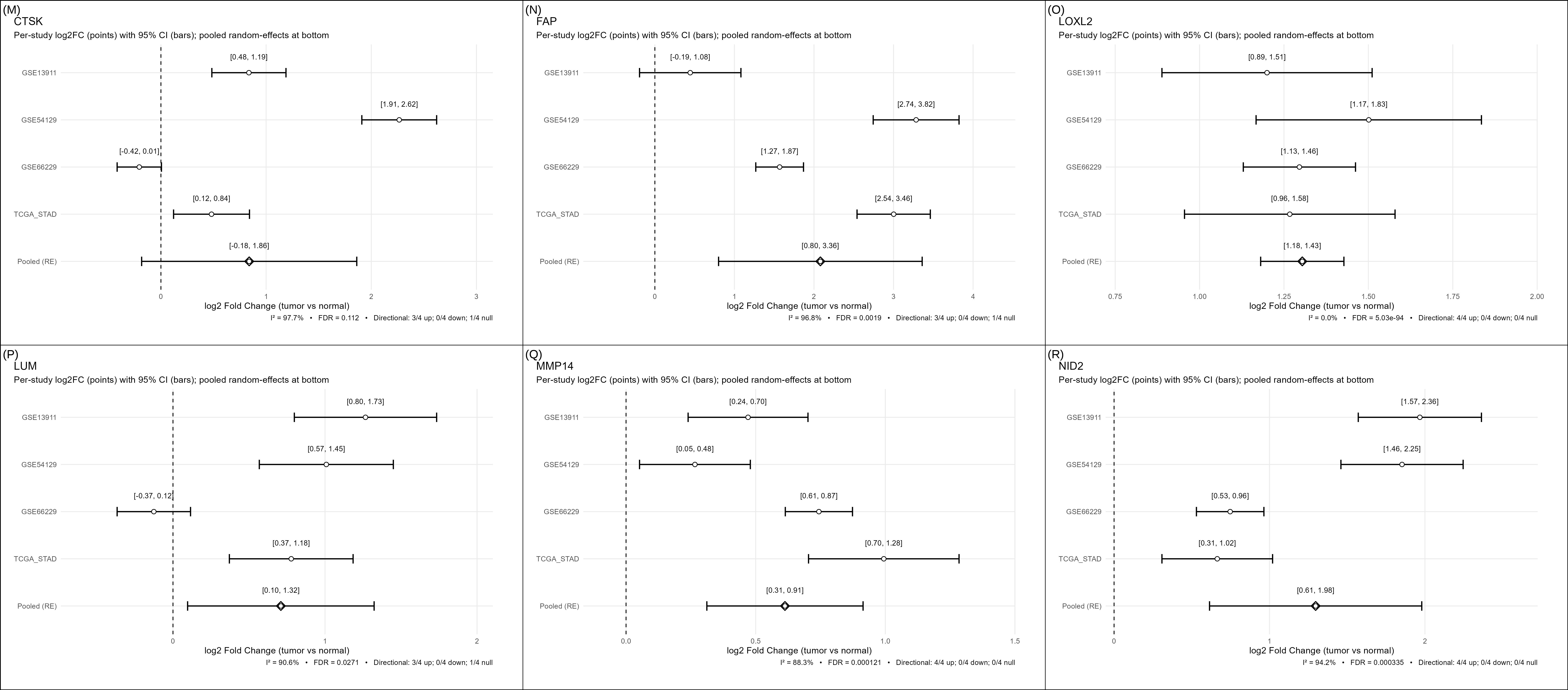


**Supplementary figure 2M-R**: Forest plots of CTSK, FAP, LOXL2, LUM, MMP14, and NID2 with I^2^, FDR and directional information


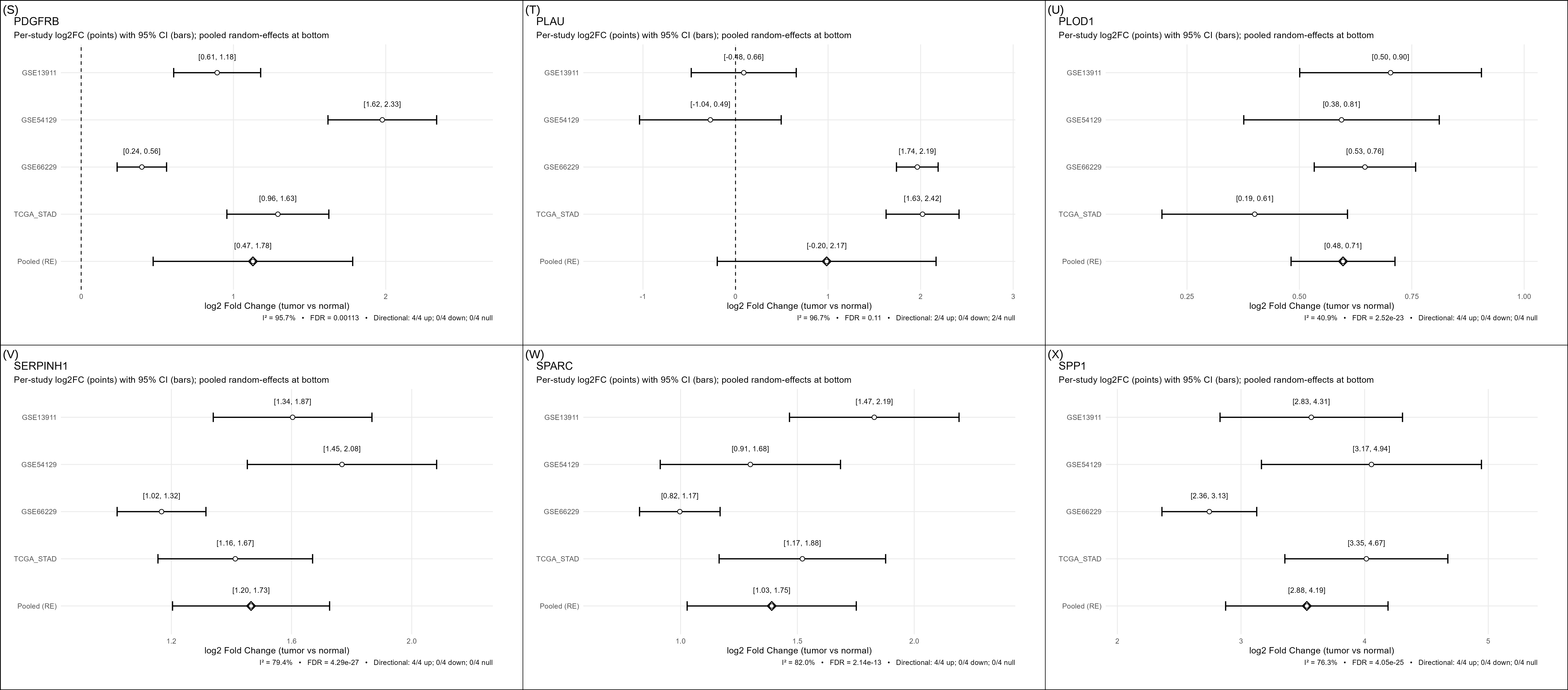


**Supplementary figure 2S-X**: Forest plots of PDGFRB, PLAU, PLOD1, SERPINH2, SPARC, and SPP1 with I^2^, FDR and directional information


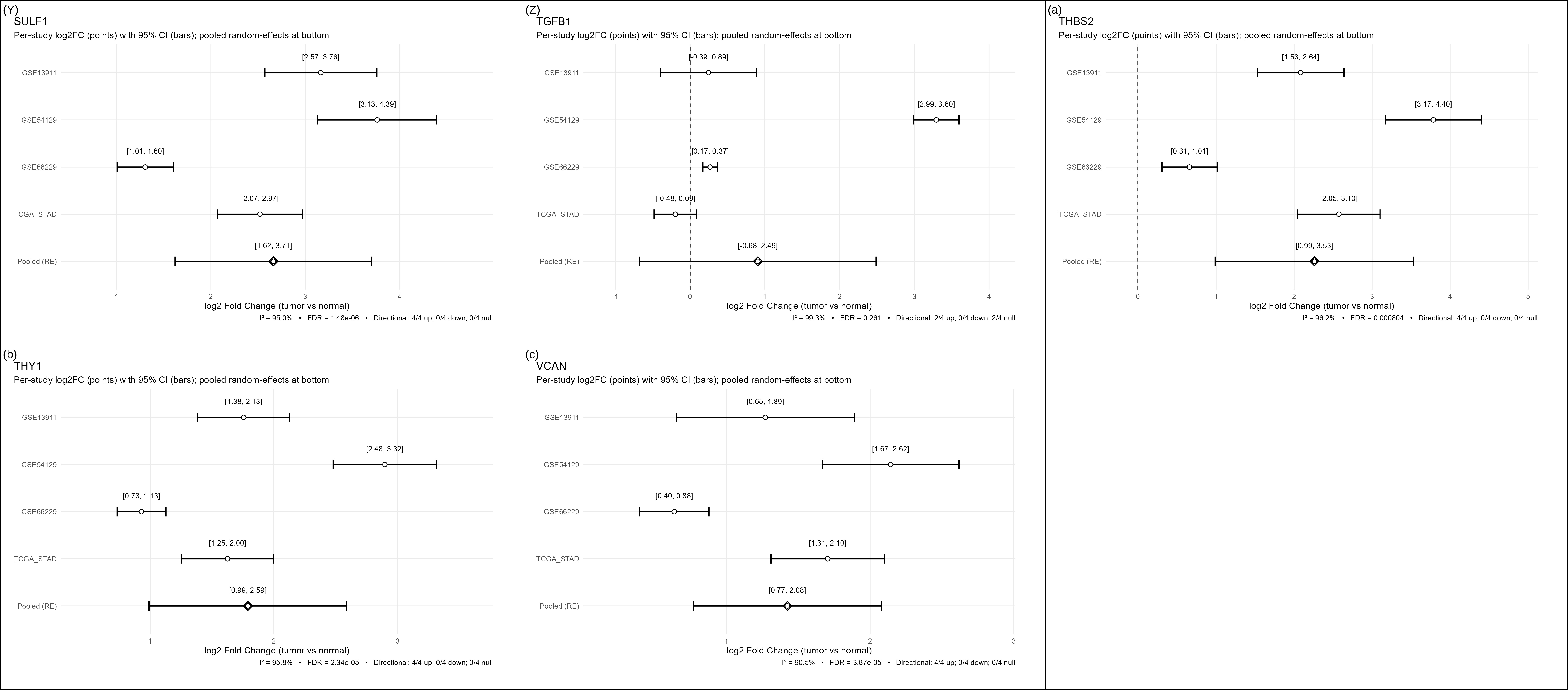


**Supplementary figure 2Y-Z and 2a-c**: Forest plots of CTSK, FAP, LOXL2, LUM, MMP14, and NID2 with I^2^, FDR and directional information
